# Intake of B vitamins and their circulating levels in relation to incident stroke in women and men: Findings from two national prospective cohorts in the United States

**DOI:** 10.1016/j.ajpc.2026.101534

**Published:** 2026-03-12

**Authors:** Xinge Zhang, Bo Yang, Bernadette Boden-Albala, Hoda Anton-Culver, Matthew Allison, Linda Van Horn, Tracy E. Madsen, Shaista Malik, JoAnn E. Manson, Marian L. Neuhouser, Alexander P. Reiner, Nathan D. Wong, Simin Liu

**Affiliations:** a Department of Epidemiology & Biostatistics, Joe C. Wen School of Population and Public Health, University of California, Irvine, CA, USA; b Center for Global Cardiometabolic Health & Nutrition, University of California, Irvine, CA, USA; c Mary and Steve Wen Cardiovascular Division, Department of Medicine, School of Medicine, University of California, Irvine, CA, USA; d Genetic Epidemiology Institute, Department of Medicine, University of California, Irvine, CA, USA; e Department of Family Medicine, University of California, San Diego, La Jolla, CA, USA; f Department of Preventive Medicine, Feinberg School of Medicine, Northwestern University, Chicago, IL, USA; g Department of Emergency Medicine, Larner College of Medicine, University of Vermont, Burlington, VT, USA; h Susan Samueli Integrative Health Institute, Mary and Steve Wen Cardiovascular Division, Department of Medicine, University of California, Irvine, CA, USA; i Department of Epidemiology, Harvard T. H. Chan School of Public Health, Boston, MA, USA; j Channing Division of Network Medicine, Department of Medicine, Brigham and Women’s Hospital and Harvard Medical School, Boston, MA, USA; k Division of Preventive Medicine, Department of Medicine, Brigham and Women’s Hospital and Harvard Medical School, Boston, MA, USA; l Division of Public Health Sciences, Fred Hutchinson Cancer Center, Seattle, WA, USA; m Department of Epidemiology, University of Washington, Seattle, WA, USA

**Keywords:** B vitamin, Folate, Stroke, Thiamin, Riboflavin, Niacin

## Abstract

**Background:**

Evidence linking different B vitamins to stroke risk remains sparse, particularly regarding long-term intake and dose-response thresholds in populations fortified with folic acids.

**Objective:**

To prospectively investigate the associations of long-term intake of B vitamins and their circulating levels with incident stroke.

**Methods:**

Using a validated food frequency questionnaire, we assessed intake of B vitamins among 121,565 participants in the Women's Health Initiative (WHI). We also examined circulating levels of B vitamins in relation to stroke risk among 99,660 All of Us Research Program (AoU) participants. Multivariable Cox models estimated hazard ratios (HRs) and their 95 % confidence intervals (CIs).

**Results:**

In WHI (6803 incident stroke cases; median follow-up: 18.4 years), higher long-term intakes of thiamin, riboflavin, niacin, pyridoxine, and folate were significantly associated with lower stroke risk, with HRs (95 % CI) of 0.84 (0.76, 0.92), 0.90 (0.81, 0.99), 0.80 (0.72, 0.88), 0.88 (0.80, 0.96), and 0.88 (0.80, 0.97) comparing the highest to the lowest quintiles, respectively. Most B vitamins exhibited reverse J-shaped associations, whereas folate showed a linear inverse relationship up to at least 2000 dietary folate equivalent (DFE)/day. In AoU (5163 incident stroke cases; median follow-up: 5.7 years), higher plasma folate and pyridoxine were associated with lower risk (HRs: 0.86 and 0.50, respectively).

**Conclusions:**

In two large prospective cohorts of US adults, higher intake of thiamin, riboflavin, niacin, pyridoxine, and folate, as well as higher circulating concentrations of pyridoxine and folate, were associated with a lower risk of stroke, even after folic acid fortification.

## 1 Introduction

Stroke is the third leading cause of death worldwide and a major contributor to long-term disability. The Global Burden of Disease study estimated 12 million new strokes and 94 million people living with stroke-related disabilities in 2021 [1]. Given the narrow therapeutic window for acute treatment [2] and the high costs of care [3], primary prevention remains the key strategy to reduce the risk of stroke and its burden.

B vitamins are a group of eight essential coenzymes critical in one-carbon metabolism and mitochondrial function for cerebrovascular health [4,5]. While folic acid (B₉), pyridoxine (B₆), and cobalamin (B₁₂) in stroke prevention have been extensively studied [[6], [7], [8]], several knowledge gaps remained. First, there is a lack of prospective data evaluating long-term B-vitamin intake and subsequent stroke risk within a post-fortification landscape. Second, much evidence for thiamin (B_1_), riboflavin (B_2_), and niacin (Niacin) is limited to cross-sectional studies, and pantothenic acid (B_5_) and biotin (B_7_) have not been studied. Third, while the importance of biological status of B vitamins is well recognized, few prospective studies have integrated both intake and circulating levels of B vitamins in assessing stroke risk. Lastly, although lowering homocysteine has been mechanistically linked to the benefits of folic acid, pyridoxine, and cobalamin, the proportion of risk reduction mediated by the homocysteine pathway remains unknown, especially given that several homocysteine-independent mechanisms have been proposed.

Using data from two large, national cohorts in the US, we therefore investigated 1) the associations and the shape of dose-response relationships of both total intakes and circulating biomarkers of B vitamins with incident stroke, 2) the interactions among these measures of B vitamins, and 3) the proportion of the B vitamin-stroke association mediated by homocysteine.

## 2 Methods

### 2.1 Study populations

This prospective analysis used data from the Women’s Health Initiative (WHI) and the National Institutes of Health All of Us Research Program (AoU). The WHI enrolled 161,808 postmenopausal women aged 50–79 years at 40 US centers between 1993 and 1998, with annual follow-up via clinic visits or questionnaires and central adjudication of cardiovascular events (including strokes) [9,10]. The AoU is an ongoing nationwide cohort launched in 2018 that integrates survey data, biospecimens, and linked retrospective electronic health records [11].

In the WHI, where multiple food-frequency questionnaire (FFQ) assessments were available, baseline was instead set to the latest date of recorded relevant B-vitamin exposure. Following the enrollment period (1993–1998) in WHI, 131,463 women (85.0 %) completed at least one subsequent dietary assessment, 45,105 (29.2 %) completed two, and 35,492 (22.9 %) completed three (Fig. S1). After excluding women with a history of stroke at baseline, those without repeated measures of B-vitamin intake, those with implausible energy intake (<600 or >5000 kcal/day), and those with missing stroke status, the final WHI analytic cohort comprised 121,565 women. For AoU, baseline was defined as the earliest date of any relevant recorded B-vitamin exposure. We excluded individuals with a history of stroke at baseline, no B-vitamin biomarker data, or missing stroke status. The AoU analytic cohort (*N* = 99,660) were extracted from the total program population (*N* = 633,544) by selecting participants with at least one available B-vitamin biomarker recorded in their linked electronic health records. Compared to participants without biomarker data, those in the analytic cohort were generally older and exhibited a higher prevalence of cardiovascular risk factors (Table S1). Each participant provided written informed consent, and both studies received approval from local institutional review boards.

### 2.2 Exposure assessment

In WHI, intake of thiamin, riboflavin, niacin, pantothenic acid, pyridoxine, folate, and cobalamin was estimated using a validated 122-item FFQ, supplemented by self-reported data on the types, frequencies, and dosages of supplement use [12]. Exposure assessment was conducted at study entry (1993–1998) and repeated through 2005. Time-weighted mean values were calculated. In AoU, serum or plasma values for thiamin, pyridoxine, pyridoxal-5′-phosphate, folate (and red blood cell [RBC] folate), cobalamin, and total homocysteine were extracted from electronic health records. Cohort-specific quintiles were derived for categorical analyses. Details are provided in the Supplement Methods.

### 2.3 Outcome ascertainment

In WHI, trained neurologists reviewed hospital records and imaging and classified strokes according to WHI criteria, defined as the rapid onset of a persistent neurological deficit attributable to cerebral infarction or hemorrhage, lasting ≥24 h or until death, and without another obvious cause [13] Ischemic strokes were attributed explicitly to cerebral infarction (ICD-9 codes 433–434), whereas hemorrhagic strokes were attributed to either intracerebral (ICD-9 codes 431) or subarachnoid hemorrhage (ICD-9 codes 430).

In AoU, stroke was identified in the electronic health records as the earliest inpatient diagnosis with an ICD-10 code I60–I64 or an ICD-9 code 430–432, 433.X1, or 434.X1. Hemorrhagic strokes were specifically identified by the ICD-10 codes I60-I62 or ICD-9 code 430–432, and ischemic strokes were identified by the ICD-10 code I63 or ICD-9 code 433.X1 and 434.X1.

Follow-up for both cohorts extended from baseline until the first incident stroke, death, withdrawal, loss to follow-up, or the administrative censoring date (September 30, 2023, for WHI and October 1, 2023, for AoU).

### 2.4 Covariates

Baseline covariates including age, sex (AoU only), race/ethnicity, education level, household income brackets (WHI only), Townsend Deprivation Index (TDI, AoU only), smoking status, alcohol consumption, physical activity measured by total Metabolic equivalent of task (MET) minutes per week in WHI, family history of stroke, body-mass index (BMI), abdominal obesity measured by waist-hip ratio (≥0.85 for women and ≥0.90 for men), ever use of oral glucose-lowering drugs, and ever use of lipid-lowering drugs. Hypertension was defined as self-reported diagnosis, use of antihypertensive medication, systolic blood pressure ≥130 mmHg, or diastolic blood pressure ≥90 mmHg. In WHI, time-weighted average intakes of total energy and a panel of non-B-vitamin nutrients (vitamins C, D, E, α- and β-carotene, selenium, magnesium, potassium, calcium, dietary fiber, sodium, and total protein) were also included. Category cut points and coding are detailed in Table 1 (Table 1A, Table 1B).

**Table 1A:** Baseline characteristics of participants free of stroke at baseline in the National Women’s Health Initiative, stratified by incident stroke status during follow-up.

Characteristic	No Incident Stroke (*N* = 114,762)	Incident Stroke (*N* = 6803)
Age at baseline, mean ± SD	62.9 ± 7.1	66.7 ± 6.5
< 60 years	39,759 (35 %)	993 (15 %)
60–69 years	52,048 (45 %)	3305 (49 %)
≥70 years	22,955 (20 %)	2505 (37 %)
Race/ethnicity, n ( %)*		
White	99,637 (87 %)	5793 (85 %)
Black	8807 (8 %)	722 (11 %)
Asian	2948 (3 %)	101 (1 %)
Hispanic/Latino	1367 (1 %)	69 (1 %)
Others	2003 (2 %)	118 (2 %)
Income, n ( %)		
<$20k	15,206 (14 %)	1165 (18 %)
$20–49.9k	47,505 (44 %)	3174 (50 %)
$50–74.9k	22,671 (21 %)	1168 (18 %)
≥$75k	22,062 (21 %)	888 (14 %)
Education, n ( %)		
Below college	65,868 (58 %)	4068 (60 %)
College degree or above	4,8093 (42 %)	2688 (40 %)
Smoking, n ( %)		
Never	58,475 (52 %)	3504 (52 %)
Past	48,264 (43 %)	2765 (41 %)
Current	6686 (6 %)	437 (7 %)
Drinking, n ( %)		
0/past drinker	31,517 (28 %)	2051 (30 %)
>0–<7 drinks/week	68,888 (60 %)	3896 (58 %)
≥7 drinks/week	13,774 (12 %)	811 (12 %)
MET minutes per week, n ( %)*		
<600	81,328 (74 %)	4850 (76 %)
≥600-<1200	16,964 (15 %)	960 (15 %)
≥1200	11,266 (10 %)	595 (9 %)
Family history of stroke, n ( %)	41,277 (38 %)	2815 (44 %)
BMI category, n ( %)		
Underweight/normal	41,449 (36 %)	2225 (33 %)
Overweight	39,473 (35 %)	2441 (36 %)
Obesity	32,876 (29 %)	2081 (31 %)
Abdominal obesity, n ( %)	30,022 (26 %)	2148 (32 %)
Hypertension, n ( %)	51,854 (45 %)	4245 (63 %)
Ever use of lipid-lowering drugs, n ( %)	14,550 (13 %)	997 (16 %)
Thiamin, mg/day, mean ± SD	6.2 ± 23.1	5.9 ± 20.4
Riboflavin, mg/day, mean ± SD	6.2 ± 18.6	6.0 ± 18.2
Niacin, mg/day, mean ± SD	42.0 ± 113.2	39.5 ± 92.2
Pantothenic acid, mg/day, mean ± SD	10.1 ± 38.7	10.1 ± 38.4
Pyridoxine, mg/day, mean ± SD	8.4 ± 31.6	8.2 ± 29.8
Folate, DFE/day, mean ± SD	820.6 ± 478.3	798.5 ± 446.4
Cobalamin, mcg/day, mean ± SD	21.8 ± 70.6	22.7 ± 77.2
Total energy intake, Kcal, mean ± SD	1587.5 ± 551.9	1575.3 ± 554.9
Vitamin C, mg/day, mean ± SD	101.9 ± 49.1	102.7 ± 48.8
Vitamin D, mcg/day, mean ± SD	4.4 ± 2.7	4.5 ± 2.8
Vitamin E, IU/day, mean ± SD	9.3 ± 5.0	9.3 ± 5.1
Alpha-carotene, mcg/day, mean ± SD	701.0 ± 470.2	703.5 ± 471.1
Beta-carotene, mcg/day, mean ± SD	3305.1 ± 1928.5	3366.6 ± 1932.1
Selenium, mcg/day, mean ± SD	89.6 ± 34.2	88.3 ± 34.4
Magnesium, mg/day, mean ± SD	253.9 ± 86.3	251.8 ± 86.9
Potassium, mg/day, mean ± SD	2615.8 ± 876.4	2602.8 ± 884.5
Calcium, mg/day, mean ± SD	817.7 ± 402.4	809.9 ± 399.1
Fiber, g/day, mean ± SD	16.1 ± 6.2	16.0 ± 6.2
Sodium, mg/day, mean ± SD	2666.6 ± 1001.7	2646.8 ± 1019.1
Total protein, g/day, mean ± SD	67.1 ± 25.2	66.4 ± 25.4

**Table 1B:** Baseline characteristics of participants free of stroke at baseline in the National Institute of Health’s All of Us, stratified by incident stroke status during follow-up.

Characteristic	No Incident Stroke (*N* = 94,497)	Incident Stroke (*N* = 5163)
Age at baseline, mean ± SD	53.5 ± 15.6	58.5 ± 13.4
< 60 years	58,788 (62 %)	2696 (52 %)
60–69 years	21,577 (23 %)	1430 (28 %)
≥70 years	14,132 (15 %)	1037 (20 %)
Sex, n ( %)		
Women	59,657 (64 %)	2782 (55 %)
Men	33,827 (36 %)	2282 (45 %)
Race/ethnicity, n ( %)*		
White	58,555 (62 %)	2884 (56 %)
Black	14,339 (15 %)	1043 (20 %)
Asian	2131 (2 %)	64 (1 %)
Hispanic/Latino	11,549 (12 %)	691 (13 %)
Others	7923 (8 %)	481 (9 %)
TDI, mean ± SD	0.3 ± 0.1	0.3 ± 0.1
Education, n ( %)		
Below college	21,699 (23 %)	1559 (31 %)
College degree or above	71,022 (77 %)	3471 (69 %)
Ever smoked 100 cigarettes during lifetime, n ( %)	39,252 (43 %)	2558 (51 %)
Drinking, n ( %)		
Never	65,502 (69 %)	3269 (63 %)
>0–<4 drinks/week	10,695 (11 %)	503 (10 %)
≥4 drinks/week	18,300 (19 %)	1391 (27 %)
Family history of stroke, n ( %)	8171 (9 %)	448 (9 %)
BMI category, n ( %)		
Underweight/normal	25,441 (28 %)	1259 (25 %)
Overweight	26,989 (29 %)	1547 (31 %)
Obesity	39,506 (43 %)	2242 (44 %)
Abdominal obesity, n ( %)	45,990 (61 %)	2988 (72 %)
Hypertension, n ( %)	27,759 (30 %)	2075 (40 %)
Ever use of oral glucose-lowering drugs, n ( %)	19,571 (21 %)	1395 (27 %)
Ever use of lipid-lowering drugs, n ( %)	36,550 (39 %)	2563 (50 %)
Plasma thiamin (nmol/L), mean ± SD*	113.7 ± 94.1	121.2 ± 144.3
Plasma pyridoxine (nmol/L), mean ± SD	96.5 ± 117.2	72.4 ± 90.5
Plasma pyridoxal-5′-phosphate (nmol/L), mean ± SD	84.3 ± 101.2	47.0 ± 42.4
Plasma folate (ng/mL), mean ± SD	20.8 ± 78.5	18.9 ± 60.5
RBC folate (ng/mL), mean ± SD	729.4 ± 323.7	712.1 ± 315.9
Plasma cobalamin (pg/mL), mean ± SD	588.4 ± 506.6	576.6 ± 441.6

DFE: dietary folate equivalent; IQR: interquartile range; MET: Metabolic equivalent of task; SD: Standard deviation; TDI: Townsend Deprivation Index; BMI: Body-mass index. *Not all covariates have complete data.

### 2.5 Statistical analysis

Analyses were conducted separately for the WHI and AoU cohorts. For each, we summarized baseline characteristics of included participants using mean (standard deviation, SD) for continuous variables and counts (percentages) for categorical variables.

For the primary analysis, we used Cox proportional hazards models to estimate hazard ratios (HRs) and 95 % confidence intervals (CIs) for incident stroke and its subtypes both across quintiles and per 1-SD increment of each B-vitamin exposure. Models were adjusted for the covariates listed above. We explored potential non-linear dose-response relationships using restricted cubic splines with knots at the 25th, 50th, and 75th percentiles. To minimize bias from extreme values, outliers were identified and addressed during model evaluation through residual diagnostics. We also assessed the association between changes in B-vitamin exposure and incident stroke risk.

To assess biological interplay, we cross-classified participants into tertiles of exposure pairs and included multiplicative interaction terms in the fully adjusted models. Tertiles were used because some biomarkers had small sample sizes. Additive interaction was quantified using the relative excess risk due to interaction (RERI). The detailed calculation process is provided in the Supplement Methods.

To investigate homocysteine-mediated effects, we limited the analysis to participants with available homocysteine measurements (*n* = 8697). We first confirmed the B-vitamin-stroke associations. For exposures that remained significant after adjustment, we then used a parametric g-formula (counterfactual framework) to estimate the proportion of the association mediated by homocysteine. CIs for the mediated proportion were generated using 1000 bootstrap replications.

We conducted subgroup analyses stratified by the baseline covariates described earlier in the Methods. Sensitivity analyses were conducted by (1) restricting to non-users of multivitamin supplements and (2) employing Fine–Gray sub-distribution models to handle death as a competing event. All analyses were performed using R (version 4.5.0). A two-sided p-value<0.05 was considered statistically significant. To address multiple comparisons, we applied the false discovery rate (FDR) procedure independently within each cohort. Results were considered statistically significant at a q-value<0.05, denoting an expected proportion of false discoveries below 5 % [14].

## 3 Results

### 3.1 Baseline characteristics

Table 1 presents baseline characteristics for WHI and AoU. The WHI cohort consisted entirely of postmenopausal women, whereas 37 % of the AoU cohort were men. Individuals with incident stroke were generally older, more often black, and had lower income and education levels. Stroke groups also had higher rates of hypertension, abdominal obesity, and lipid-lowering drug use.

### 3.2 Total B-vitamin intake and incident stroke in WHI

**Fig. 1:**
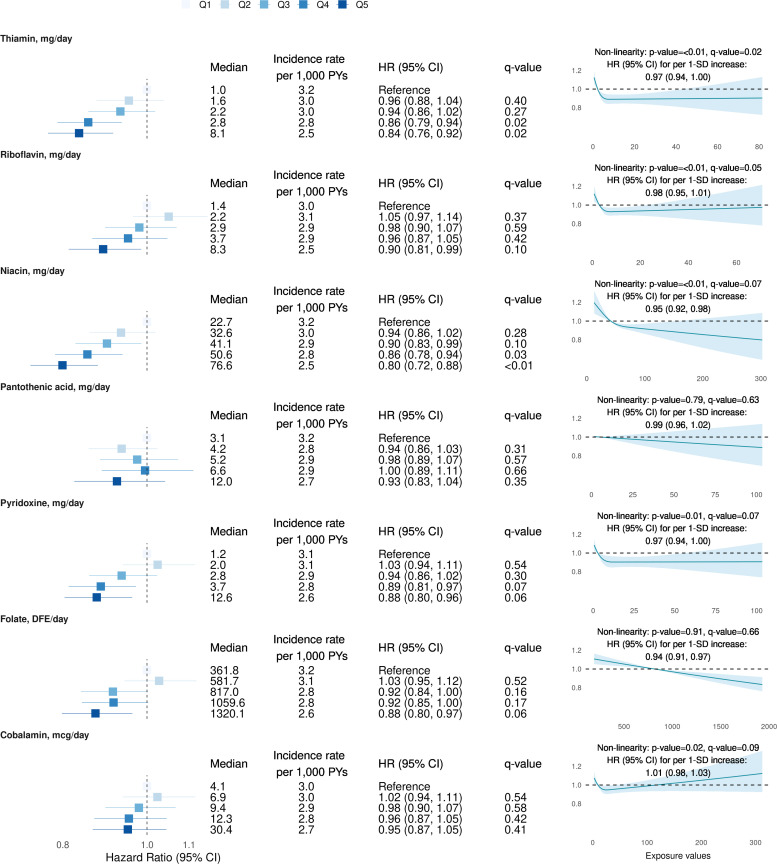
Associations of long-term intakes of individual B-vitamins with incident stroke in the Women’s Health Initiatives. Hazard ratios and 95 % confidence intervals are shown for quintile comparisons and per 1-standard deviation increase in circulating biomarker concentration. Models were adjusted for age, ethnicity, income, education, smoking, drinking, MET-mins per week, family history of stroke, BMI group, abdominal obesity, hypertension, ever use of lipid-lowering drugs, time-weighted average intakes of total energy, vitamins C, D, E, α- and β-carotene, selenium, magnesium, potassium, calcium, dietary fiber, sodium, and total protein. CI: confidence interval; DFE: dietary folate equivalent; HR: hazard ratios.

**Fig. 2:**
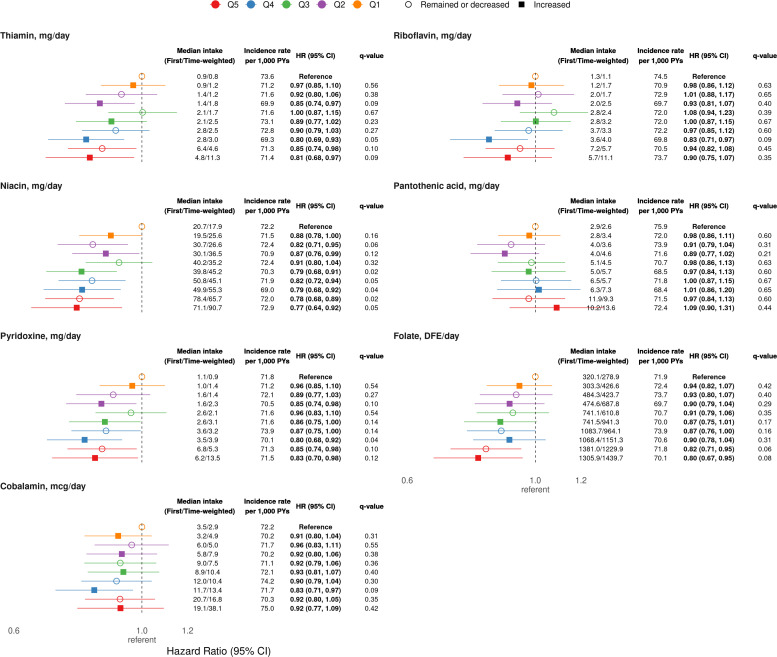
Comparisons of stroke risk across quintiles of baseline intakes and subsequent changes in individual B-vitamins in the Women’s Health Initiatives. Models were adjusted for age, ethnicity, income, education, smoking, drinking, MET-mins per week, family history of stroke, BMI group, abdominal obesity, hypertension, ever use of lipid-lowering drugs at baseline, time-weighted average intakes of total energy, vitamins C, D, E, α- and β-carotene, selenium, magnesium, potassium, calcium, dietary fiber, sodium, and total protein. CI: confidence interval; DFE: dietary folate equivalent; HR: hazard ratios.

Over a median follow-up of 18.4 years (interquartile range [IQR] 8.0–23.8 years) in the WHI cohort, 6803 incident strokes (3621 ischemic and 674 hemorrhagic) occurred. After adjustment for known stroke risk factors, higher daily intakes of thiamin, niacin, pyridoxine, and folate were each significantly associated with lower stroke risk. Comparing the highest (Q5) to the lowest (Q1) intake quintile, the HRs with 95 % CIs were 0.84 (0.76, 0.92) for thiamin (Q5 vs. Q1 median intake: 8.1 mg/day vs. 1.0 mg/day), 0.80 (0.72, 0.88) for niacin (76.6 mg/day vs. 22.7 mg/day), 0.88 (0.80, 0.96) for pyridoxine (12.6 mg/day vs. 1.2 mg/day), and 0.88 (0.80, 0.97) for folate (1320.1 dietary folate equivalent [DFE]/day vs 361.8 DFE/day) (Fig. 1). Furthermore, increasing long-term exposure relative to the baseline level was consistently associated with greater risk reduction, independent of baseline intake levels (Fig. 2). For thiamin, riboflavin, niacin, and pyridoxine, the dose-response relationship with stroke risk was reverse J-shaped. In contrast, folate exhibited a clear linear inverse association up to at least 2000 DFE/day (Fig. 1). Adjusted HRs per 1-SD higher intake were 0.97 (95 % CI: 0.94, 1.00) for thiamin, 0.98 (0.95, 1.01) for riboflavin, 0.95 (0.92, 0.98) for niacin, 0.97 (0.94, 1.00) for pyridoxine, and 0.94 (0.91, 0.97) for folate. These associations remained for ischemic stroke but not for hemorrhagic stroke (Figs. S2-S3).

### 3.3 Circulating B vitamin and incident stroke

**Fig. 3:**
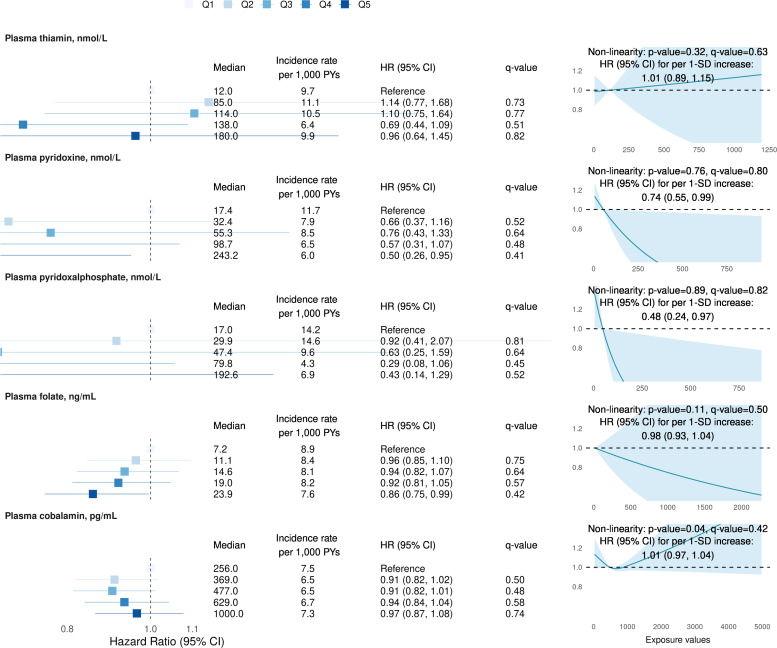
Associations of circulating concentrations of individual B-vitamins with incident stroke in the All of Us Research Program. Hazard ratios and 95 % confidence intervals are shown for quintile comparisons and per 1-standard deviation increase in circulating biomarker concentration. Models were adjusted for age, sex, ethnicity, education, smoking, drinking, family history of stroke, BMI group, abdominal obesity, hypertension, ever use of oral glucose-lowering drugs, and ever use of lipid-lowering drugs at baseline. CI: confidence interval; HR: hazard ratios.

In the AoU cohort, 5163 incident strokes (4404 ischemic and 1383 hemorrhagic) were recorded over a median follow-up of 5.7 years (IQR 2.8–9.7 years). High plasma levels of pyridoxine and folate were associated with a lower stroke risk (Fig. 3). Compared to Q1, the most substantial benefit appeared in Q5 for plasma pyridoxine (median value: 243.2 nmol/L vs 17.4 nmol/L, HR with 95 % CI: 0.50 [0.26, 0.95]) and Q5 for plasma folate (median value: 23.9 ng/mL vs 7.2 ng/mL, HR with 95 % CI: 0.86 [0.75, 0.99]), displaying a linear relationship for both (p-values for non-linearity: 0.41 for pyridoxine and 0.42 for folate). Fully adjusted HRs per 1-SD higher intake were 0.74 (95 % CI: 0.55, 0.99) for plasma pyridoxine and 0.98 (0.93, 1.04) for plasma folate. Similar associations were observed for ischemic stroke (Figs. S4–S5).

### 3.4 Mediation by homocysteine

**Fig. 4:**
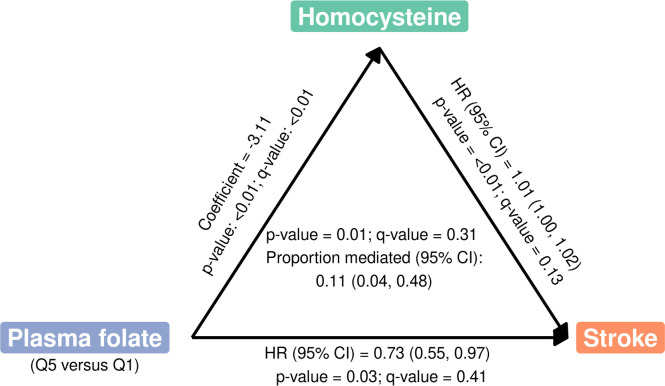
Mediation of the plasma folate–stroke association by homocysteine in All of US Research Program. All paths were adjusted for age, sex, ethnicity, education, smoking, drinking, family history of stroke, BMI group, abdominal obesity, hypertension, ever use of oral glucose-lowering drugs, and ever use of lipid-lowering drugs at baseline.

Among participants with available plasma homocysteine (mean ± SD: 11.1 ± 6.7 mmol/L), only plasma folate showed a significant inverse association with incident stroke (Fig. S6). Lower homocysteine levels accounted for 11 % (95 % CI 4 %, 48 %) of the observed protective association between plasma folate and stroke risk (Fig. 4).

### 3.5 Interaction between B vitamins, subgroup and sensitivity analyses

No significant interactions were found among B-vitamin exposures (Figs. S7-S8). In subgroup analyses, inverse associations for intakes of niacin and folate were more pronounced in younger participants (p-values for interaction = 0.01) while intakes of thiamin, niacin, and folate showed stronger associations among lipid-lowering drug users (p-values for interaction: <0.01–0.04, Fig. S9-S10). In sensitivity analyses, restricting to participants without records of multivitamin supplement use suggested attenuated but remained associations compared to the primary analysis (Fig. S11). All primary findings were robust in Fine–Gray competing-risk models (Fig. S12-S13).

## 4 Discussion

In two large, high-quality prospective cohorts of US men and women, long-term exposures to several B vitamins, including thiamin, riboflavin, niacin, pyridoxine, and folate, were significantly predictive of stroke risk over up to 20 years of follow-up. Notably, even with mandatory US folic acid fortification fully implemented by 1998, higher folate intake and plasma concentrations remained predictive of reduced stroke risk. Long-term folate intake showed a clear linear inverse relationship with stroke risk up to at least 2000 DFE/day, with no plateau or threshold observed.

The inverse associations between high folate intake and plasma folate levels and stroke risk in these two large prospective US cohorts are generally consistent with previous studies but offer new insights in the absence of an established optimal dietary intake level for stroke prevention in fortified populations. The median folate intake in our highest baseline quintile was comparable to the threshold (0.8 mg/day or 1360 DFE/day) widely cited from meta-analyses comparing high pharmacological doses (e.g., 2.5 mg/day) [6,7]. However, even within this already high stratum, higher long-term folate exposure during follow-up was associated with further reductions in stroke risk. Our dose-response analyses revealed a clear linear inverse association between long-term folate intake and stroke risk up to at least 2000 DFE/day, with no evidence of a plateau. This suggests that, within the observed range, incremental benefit may be possible beyond trial-tested supplemental doses in the post-fortification era. Whether a true plateau exists at even higher intake levels in populations with mandatory folic acid fortification, however, requires further investigation in studies with a broader range of exposures. Our mediation analysis estimated that homocysteine lowering accounts for 11 % of the plasma folate–stroke association. This aligns with trials such as HOPE-2 [15] and NORVIT [16], in which B vitamins lowered homocysteine concentrations but did not reduce CVD risk [8]. In addition, the CSPPT reported a reduction in stroke risk with folic acid, irrespective of baseline homocysteine levels [17]. Taken together, the protective effects of plasma folate appear to operate partly through homocysteine-independent pathways [[18], [19], [20]]. In contrast to the linear benefit of intake, RBC folate showed a suggestive U-shaped association in the subsample with homocysteine available. This non-linearity highlights that excessively high circulating folate could be counterproductive, perhaps due to the masking of B12 deficiency or oxidative stress induced by excessive folic acid [21,22]. These findings may also be influenced by confounding from supplementation patterns, necessitating a cautious approach to defining 'optimal' RBC folate levels in fortified populations.

Our study provides the first prospective evidence that higher dietary intakes of thiamin, riboflavin, and niacin are associated with a lower risk of stroke. These findings extend prior knowledge beyond cross-sectional associations [23], demonstrating that increased intake of these vitamins is prospectively linked to reduced stroke incidence, even among individuals with high baseline levels.

Dietary niacin intake observed in our study was modest relative to the pharmacologic doses used in trials such as AIM-HIGH [24] and HPS2-THRIVE [25], which failed to demonstrate cardiovascular benefit from niacin supplementation. It is possible that the inverse association observed may reflect broader dietary patterns or overall diet quality rather than an isolated effect of niacin itself. This interpretation is consistent with emerging metabolomic evidence linking excess niacin metabolites to inflammation [26], suggesting that the relationship between niacin and cardiovascular outcomes may differ across the intake spectrum. Moreover, the observed reverse J-shaped associations for these B vitamins-CVD relations also suggest a potential thresholds of benefit. These non-linear patterns need to be considered in designing future intervention trials and would require further investigation in additional large and high-quality prospective cohorts.

Participants in the highest intake categories are more likely to be supplement users, a group that typically demonstrates 'healthy-user' behaviors, such as higher healthcare engagement and superior diet quality. We adjusted for these factors and found consistent results in sensitivity analyses even after excluding supplement users. Further, we did observe consistent inverse associations with stroke risk for both intake and plasma levels of pyridoxine [27,28]. In contrast, cobalamin did not appear to be associated with stroke risk, consistent with findings from prior studies [28].

Beyond individual associations, we observed stronger associations of B-vitamin intake with stroke among younger participants and lipid-lowering users, consistent with previous reports [8,29,30]. However, it is important to interpret these subgroup findings with caution, as they did not remain significant after correction for multiple comparisons and should therefore be considered hypothesis-generating only, requiring confirmation in future dedicated studies. Moreover, a more substantial magnitude of relation with ischemic stroke was observed than hemorrhagic stroke, which is also consistent with prior trials indicating that folic acid supplementation reduces ischemic, but not hemorrhagic, stroke risk [17].

The following implications pertain to public health and clinical research. First, the linear inverse relationship between long-term folate intake and stroke risk, observed up to at least 2000 DFE/day without a plateau, extends and complements supplementation trials that suggested benefits may plateau at 0.8 mg/day using high-dose comparators, such as the US-based WAFACS [31], Physicians’ Health Study II [32], and COSMOS trials [7,33]. This indicates that, within the broad range of dietary exposure achievable through a fortified system, there remains significant potential for additional stroke reduction. However, these findings should not be interpreted as supporting indiscriminate increases in folate intake above recommended levels in fortified populations. The observed suggestive U-shaped association for RBC folate underscores the need for caution. Identifying the optimal intake range, one that maximizes cardiovascular benefit while minimizing harm, should be a priority for future investigation. Second, by providing the first prospective evidence for thiamin, riboflavin, and niacin, we move beyond previous cross-sectional snapshots to establish a longitudinal protective link. However, for niacin specifically, this association should be interpreted within the context of overall dietary patterns rather than as evidence of an independent nutrient-specific effect distinct from high-dose pharmacological trials. Thirdly, because homocysteine-lowering explains only a portion of the plasma folate-stroke association, elucidating homocysteine-independent pathways is crucial for designing next-generation randomized intervention trials. Finally, the observed variation in B-vitamin benefits for stroke prevention across populations highlights an important avenue for future investigation, particularly regarding the potential for targeted strategies in younger individuals and patients on lipid-lowering therapies.

There are additional issues that need to be considered when interpreting findings from these two national cohorts. First, participants with measures of B biomarkers were older and had a higher prevalence of metabolic comorbidities compared to those without biomarkers, which may have underestimated the underlying relations between these B vitamins and stroke risk in the entire AoU cohort. Second, the WHI findings are inherently restricted to postmenopausal women, although in AoU we observed similar and consistent findings in both men and women. Third, stroke ascertainment differed between the two cohorts, utilizing central adjudication in WHI and ICD codes in AoU. While ICD-based identification is more prone to misclassification (higher sensitivity but lower specificity), the consistency of our findings across these two independent methodologies suggests the observed associations are robust to differences in outcome definition.

## 5 Conclusions

In two large cohorts of US adults, higher intake and circulating levels of several B vitamins were prospectively associated with lower stroke risk, particularly for ischemic stroke. This study provides some direct evidence in support of the potential benefits of thiamin, riboflavin, and niacin for stroke prevention. Further, folate intake was prospectively associated with incrementally lower risk up to at least 2000 DFE/day, even in the post-fortification era. While these observational findings contribute to the evidence base for B vitamins in stroke prevention, findings from existing randomized trial in fortified populations remain mixed. Further research, including large and high-quality intervention trials in both fortified and unfortified populations, is warranted to determine the balance of benefits or risks concerning B-vitamin intake and establish their optimal intake as one primary strategy for stroke prevention.

**Figure:**
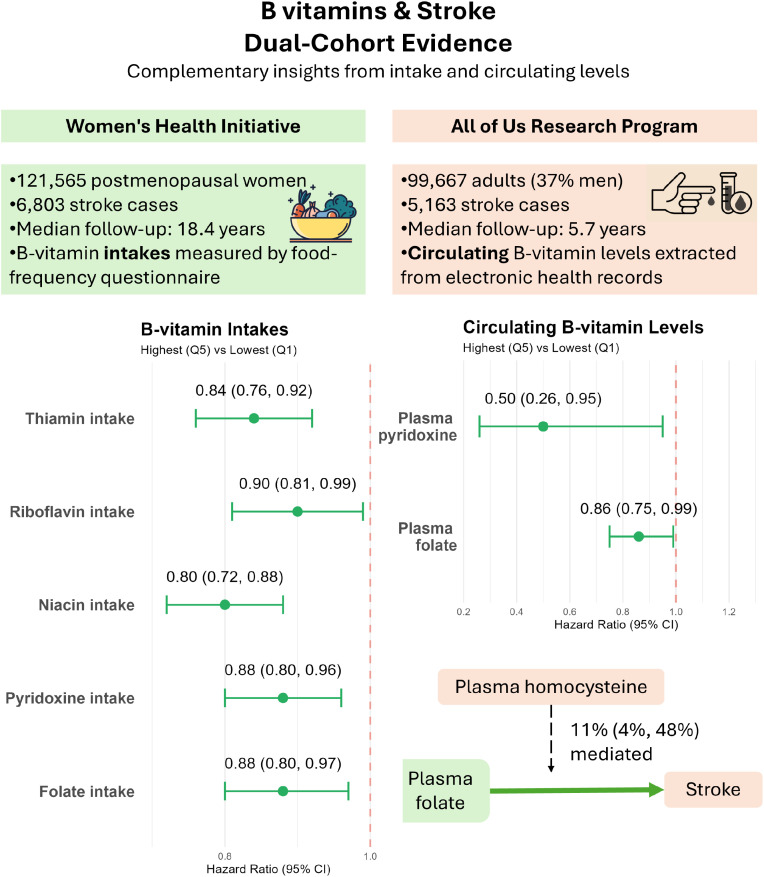
Central Illustration.

## Data statement

Anonymized data from the WHI and All of Us Research Program not published within this article will be made available by request from any qualified investigator through the programs' official access portals (WHI: https://www.whi.org/datasets; All of Us: https://www.researchallofus.org/).

## Funding sources

Drs. Simin Liu and Tracy Madsen were partly funded by R01HL164485 from the National Heart, Lung, and Blood Institute. All authors have reported that they have no relationships relevant to the contents of this paper to disclose.

## CRediT authorship contribution statement

**Xinge Zhang:** Writing – review & editing, Writing – original draft, Visualization, Validation, Methodology, Investigation, Formal analysis, Data curation, Conceptualization. **Bo Yang:** Writing – review & editing, Resources, Methodology, Data curation, Conceptualization. **Bernadette Boden-Albala:** Writing – review & editing, Methodology. **Hoda Anton-Culver:** Writing – review & editing, Resources, Methodology. **Matthew Allison:** Writing – review & editing, Methodology. **Linda Van Horn:** Writing – review & editing, Methodology. **Tracy E. Madsen:** Writing – review & editing, Methodology. **Shaista Malik:** Writing – review & editing, Methodology. **JoAnn E. Manson:** Writing – review & editing, Methodology. **Marian L. Neuhouser:** Writing – review & editing, Methodology. **Alexander P. Reiner:** Writing – review & editing, Methodology. **Nathan D. Wong:** Writing – review & editing, Methodology. **Simin Liu:** Writing – review & editing, Supervision, Resources, Methodology, Data curation, Conceptualization.

## Declaration of competing interest

The authors declare that they have no known competing financial interests or personal relationships that could have appeared to influence the work reported in this paper.

## Appendix Supplementary materials

Image, application 1
