# Supplementary material for: Intake of B vitamins and their circulating levels in relation to incident stroke in women and men: Findings from two national prospective cohorts in the United States
Source: Am J Prev Cardiol. 2026 Mar 12;28:101534. doi: 10.1016/j.ajpc.2026.101534 (PMC13325978; doi:10.1016/j.ajpc.2026.101534)

Supplement Methods

*Exposure measurements*

In Women’s Health Initiatives (WHI), a validated 122-item food-frequency questionnaire (FFQ) captured habitual consumption of foods during the previous three months, while dietary supplement use was assessed separately using a self-administered questionnaire that collected data on types of supplements taken along with their frequencies and dosages to calculate average daily nutrient intakes from these sources for 23 nutrients.^1^ Daily nutrient intake from foods was calculated using the University of Minnesota Nutrient Data System for Research.^2,3^ The correlation coefficients between the FFQ and food records ranged from 0.52 to 0.58 for B-vitamins,^2^ which indicates an acceptable relative ranking of intakes.^4^ Vitamin B12 was an exception, with a low correlation of 0.18.^2^ Total intake of thiamin, riboflavin, niacin, pantothenic acid, pyridoxine, folate, and cobalamin were estimated by summing contributions from food and supplement sources, while for folate, total intake was calculated in dietary folate equivalents (DFE) as mcg food folate + 1.7 × mcg folic acid from supplements to account for the higher bioavailability of synthetic folic acid.

In WHI, the FFQ was administered to all participants during enrollment from 1993–1998 in both the Dietary Modification (DM) trial (about 48,835 women) and the Observational Study (OS) (about 93,676 women). In the DM trial, it was collected from 100% of participants at year 1, followed by annual administration to rotating subsamples of approximately 33% through years 2–9 to monitor dietary changes and adherence. In the OS, the FFQ was given to 100% at year 3 for follow-up, with no further routine collections for the full cohort up to year 9, though small subsamples were used in later validation studies.

In AoU, historic laboratory data from electronic health records, including pre-enrollment measurements, are incorporated and harmonized via OMOP CDM, allowing some biomarkers to predate the 2018 launch. Serum or plasma values for thiamin, pyridoxine, pyridoxal-5′-phosphate, folate (and red blood cell [RBC] folate), cobalamin, and total homocysteine were extracted. Cohort-specific quintiles were derived for categorical analyses. These values derived from routine clinical laboratory assays at major US providers: chemiluminescent or electrochemiluminescent immunoassays for cobalamin and folate, liquid chromatography-tandem mass spectrometry for thiamin and pyridoxal-5′-phosphate, and enzymatic methods for homocysteine. Fasting status was unavailable, though not routinely required for B-vitamins except preferably for homocysteine.

*Estimation of relative excess risk due to interaction*

Let the joint tertile category be $(A_{i},B_{j})$ where $i$, $j$=1, 2, 3 and the reference category is the joint lowest tertile$(A_{1},B_{1})$ so that ${HR}_{11}$=1. From the fully adjusted model we obtained the HRs for each combination. RERI for cell (A_i_, B_j_, i and j =1, 2, 3) is

$${RERI}_{ij}={HR}_{ij}-{HR}_{i1}-{HR}_{1j}+1$$

A positive RERI signals attenuation of protection if one of ${HR}_{i1}$ and ${HR}_{1j}$ <1, a negative RERI means synergistic protection when both ${HR}_{i1}$ and ${HR}_{1j}$ <1.

1. Patterson RE, Levy L, Tinker LF, Kristal AR. Evaluation of a simplified vitamin supplement inventory developed for the Women's Health Initiative. *Public Health Nutr.* 1999;2(3):273-276.

2. Patterson RE, Kristal AR, Tinker LF, Carter RA, Bolton MP, Agurs-Collins T. Measurement Characteristics of the Women’s Health Initiative Food Frequency Questionnaire. *Annals of Epidemiology.* 1999;9(3):178-187.

3. Jain M, Howe GR, Rohan T. Dietary Assessment in Epidemiology: Comparison of a Food Frequency and a Diet History Questionnaire with a 7-Day Food Record. *American Journal of Epidemiology.* 1996;143(9):953-960.

4. Lombard MJ, Steyn NP, Charlton KE, Senekal M. Application and interpretation of multiple statistical tests to evaluate validity of dietary intake assessment methods. *Nutr J.* 2015;14:40.

Figure S1. Longitudinal changes in the distribution of B-vitamin intake in Women’s Health Initiative.


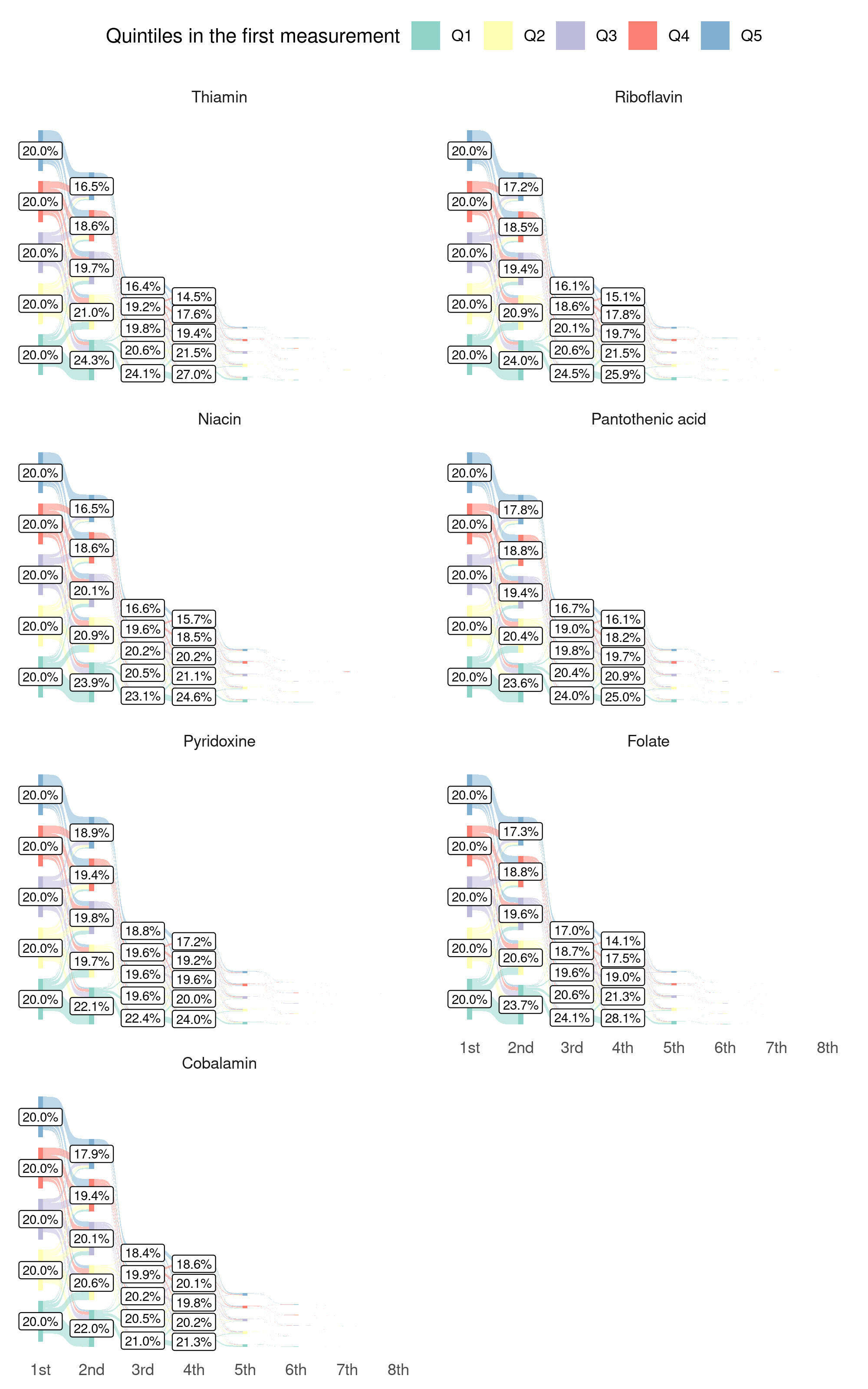


Figure S2. Associations of long-term intakes of individual B-vitamins with incident ischemic stroke in the Women’s Health Initiatives. Models were adjusted for age, ethnicity, income, education, smoking, drinking, MET-mins per week, family history of stroke, BMI group, abdominal obesity, hypertension, ever use of lipid-lowering drugs, time-weighted average intakes of total energy, vitamins C, D, E, α- and β-carotene, selenium, magnesium, potassium, calcium, dietary fiber, sodium, and total protein. CI: confidence interval; DFE: dietary folate equivalent; HR: hazard ratios.


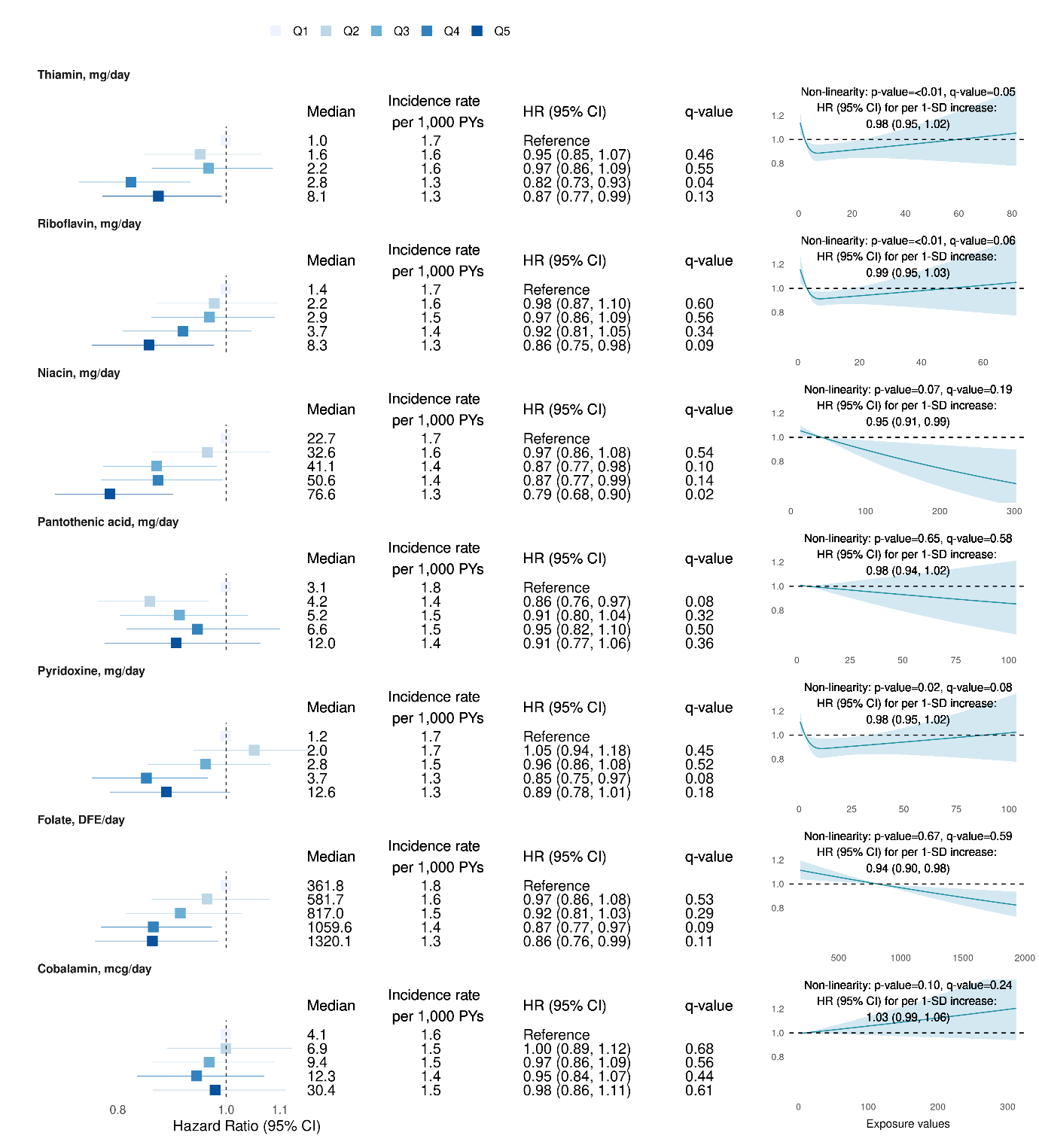


Figure S3. Associations of total intakes of individual B-vitamins with incident hemorrhagic stroke in the Women’s Health Initiatives. Models were adjusted for age, ethnicity, income, education, smoking, drinking, MET-mins per week, family history of stroke, BMI group, abdominal obesity, hypertension, ever use of lipid-lowering drugs, time-weighted average intakes of total energy, vitamins C, D, E, α- and β-carotene, selenium, magnesium, potassium, calcium, dietary fiber, sodium, and total protein. CI: confidence interval; DFE: dietary folate equivalent; HR: hazard ratios.


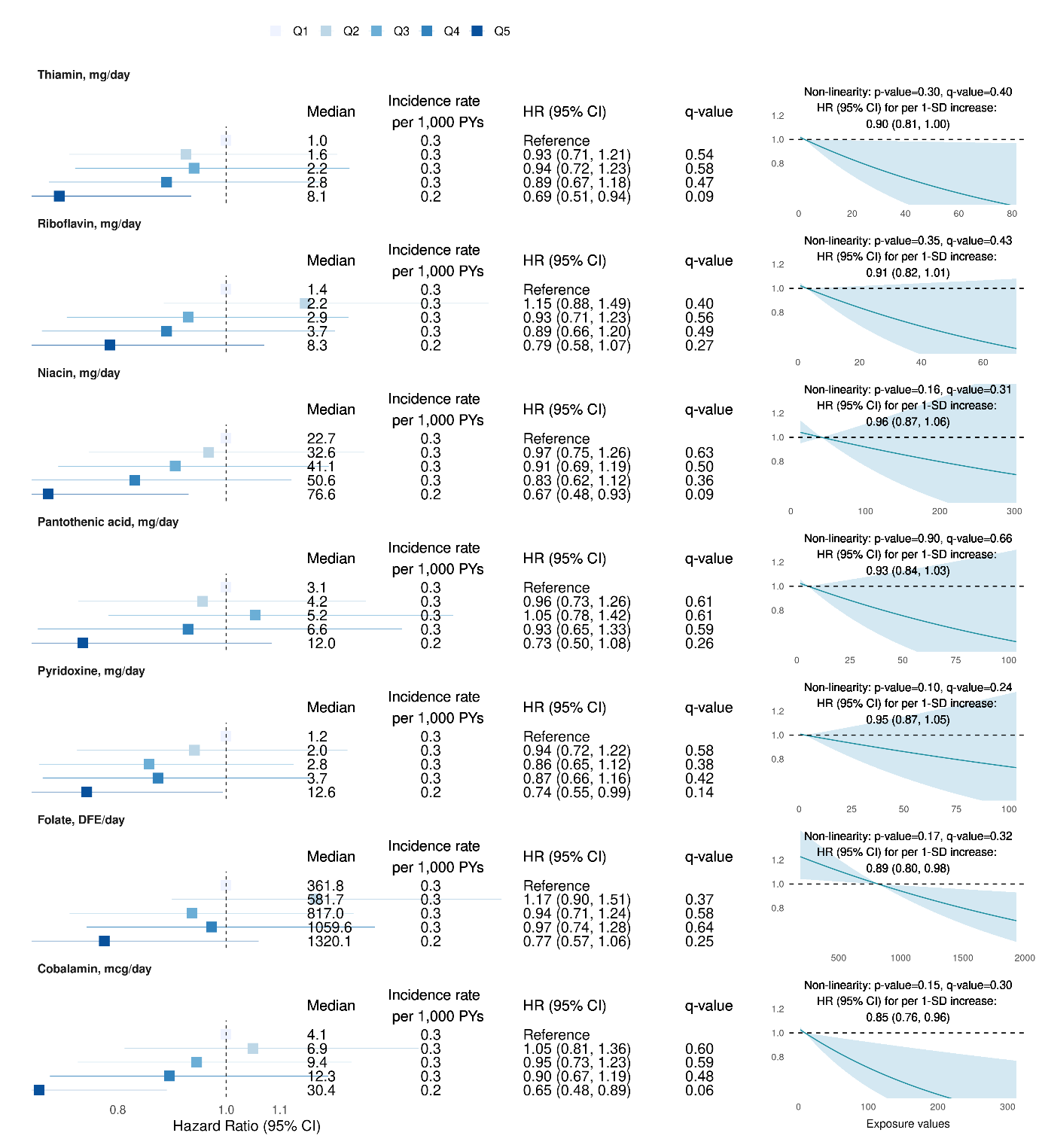


Figure S4. Associations of circulating concentrations of individual B-vitamins with incident ischemic stroke in the All of US Research Program. Models were adjusted for age, sex, ethnicity, education, smoking, drinking, family history of stroke, BMI group, abdominal obesity, hypertension, and ever use of lipid-lowering drugs at baseline. CI: confidence interval; HR: hazard ratios.


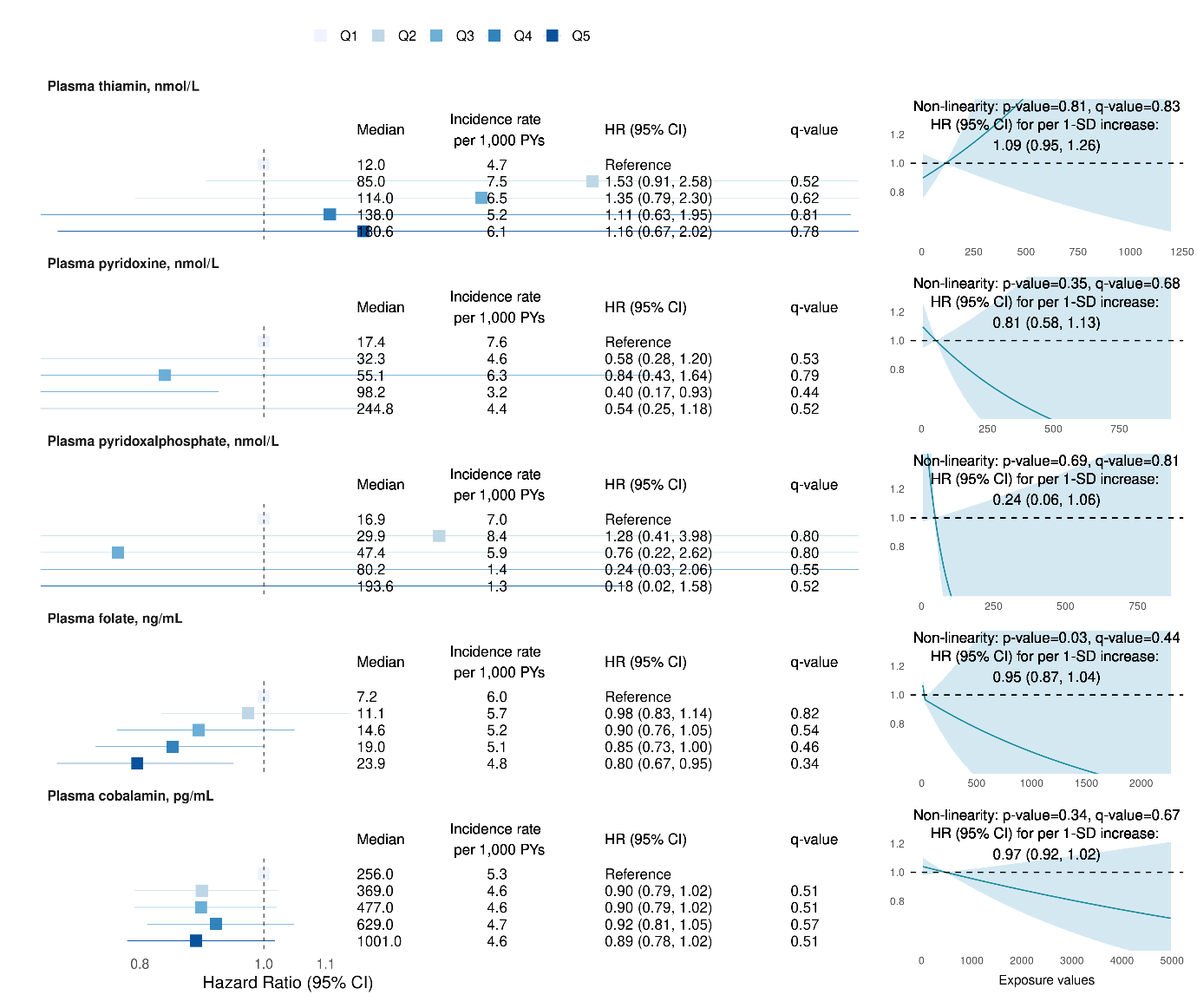


Figure S5. Associations of circulating concentrations of individual B-vitamins with incident hemorrhagic stroke in the All of US Research Program. Models were adjusted for age, sex, ethnicity, education, smoking, drinking, family history of stroke, BMI group, abdominal obesity, hypertension, ever use of oral glucose-lowering drugs, and ever use of lipid-lowering drugs at baseline. CI: confidence interval; HR: hazard ratios.


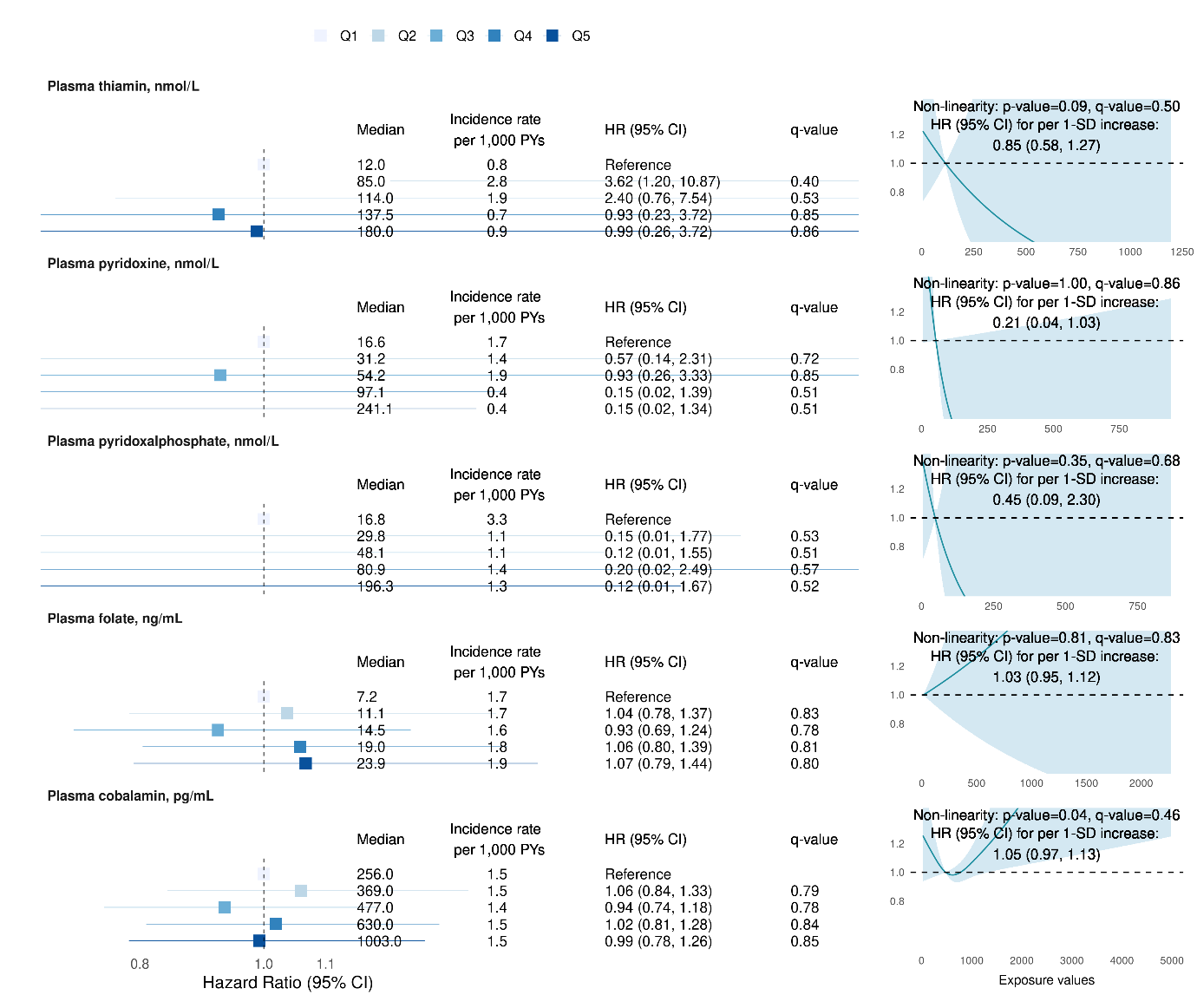


Figure S6. Associations of plasma concentrations of pyridoxine, folate, and cobalamin with incident stroke among individuals with available data on homocysteine in the All of US Research Program. Models were adjusted for age, ethnicity, income, education, total energy intake, smoking, drinking, MET-mins per week, family history of stroke, BMI group, abdominal obesity, hypertension, and ever use of lipid-lowering drugs at baseline. CI: confidence interval; HR: hazard ratios.


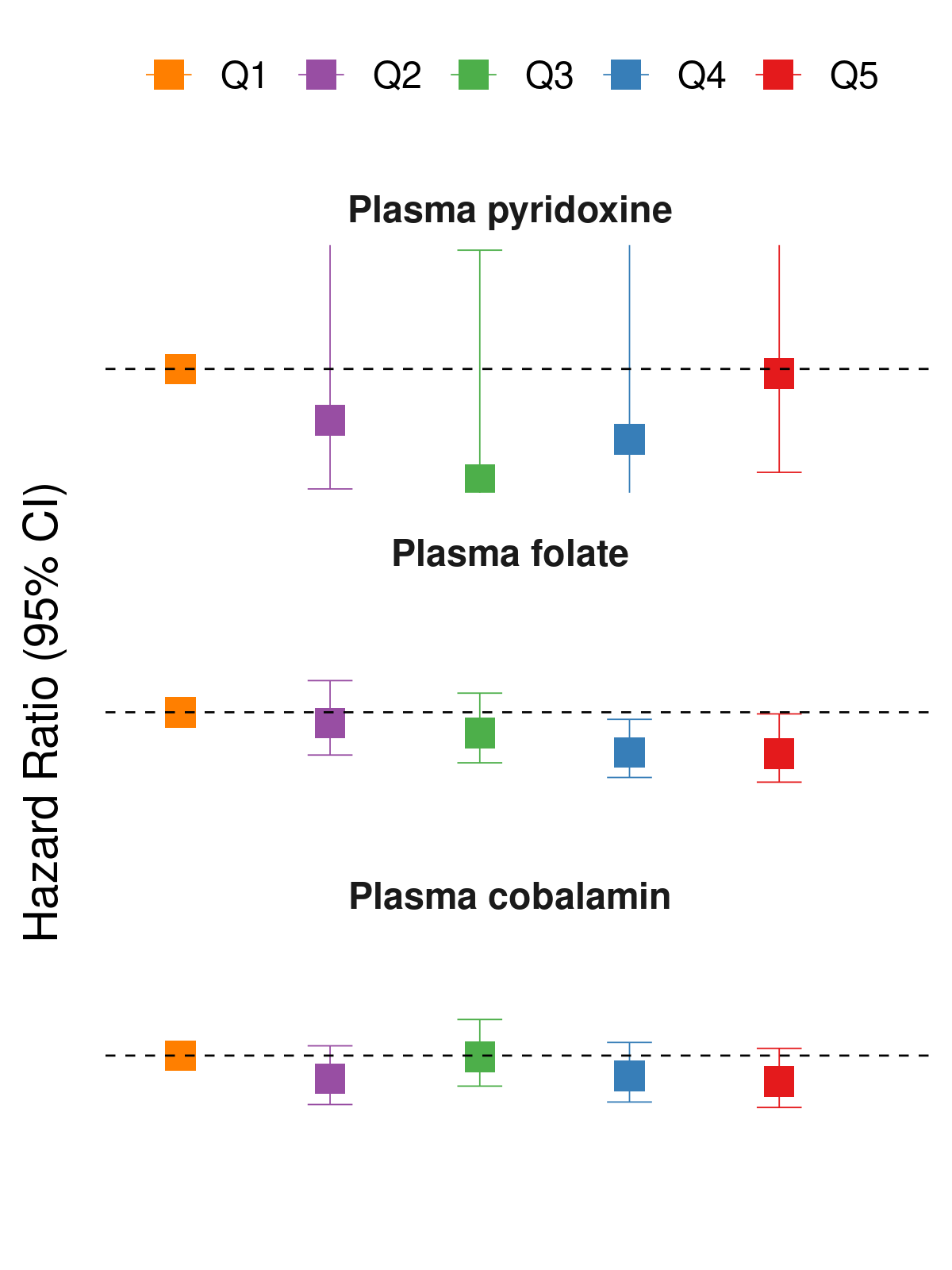


Figure S7. Joint effects of circulating B-vitamin biomarkers on incident stroke in All of US Research Program. Each heat-map cell gives the multivariable-adjusted hazard ratio (HR) and relative excess risk due to interaction (RERI) for a specific tertile (Q1–Q3) of the vitamin listed in the row, within the tertile of the vitamin shown in the column. The bottom-left cell (row Q1, column Q1) is the reference category. Cells with a black frame highlight HRs and RERIs that reach statistical significance. Models were adjusted for age, sex, ethnicity, education, smoking, drinking, family history of stroke, BMI group, abdominal obesity, hypertension, ever use of oral glucose-lowering drugs, and ever use of lipid-lowering drugs at baseline. A positive RERI signals attenuation of protection if either of the stratum-specific hazard ratios is less than 1, while a negative RERI indicates synergistic protection when both stratum-specific hazard ratios are less than 1.


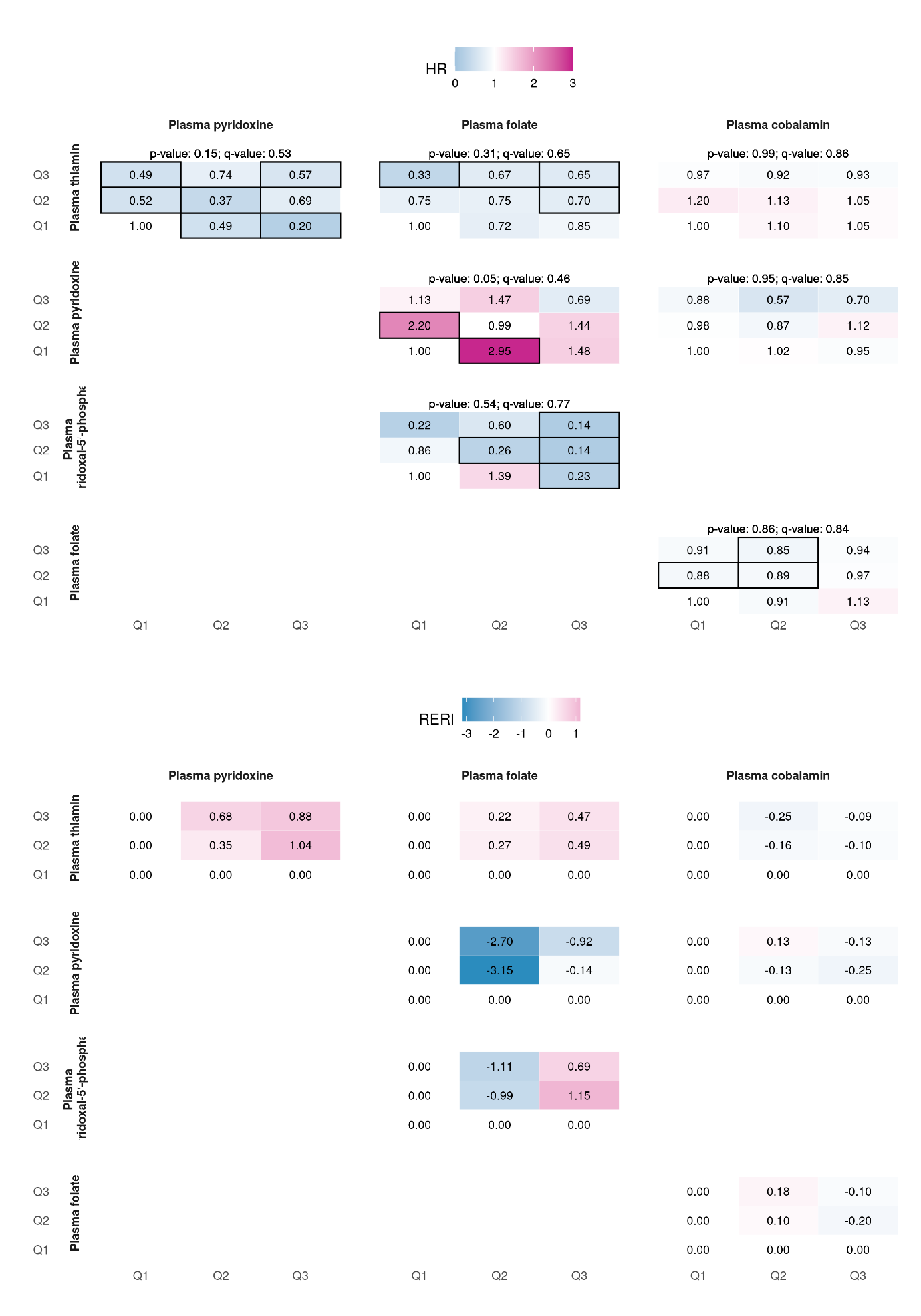


Figure S8. Joint effects of long-term B-vitamin intakes on incident stroke in Women’s Health Initiative. Each heat-map cell gives the multivariable-adjusted hazard ratio (HR) and relative excess risk due to interaction (RERI) for a specific intake tertile (Q1–Q3) of the vitamin listed in the row. The bottom-left cell (row Q1, column Q1) is the reference category. Cells with a black frame highlight HRs and RERIs that reach statistical significance. Models were adjusted for age, ethnicity, income, education, smoking, drinking, MET-mins per week, family history of stroke, BMI group, abdominal obesity, hypertension, ever use of lipid-lowering drugs, time-weighted average intakes of total energy, vitamins C, D, E, α- and β-carotene, selenium, magnesium, potassium, calcium, dietary fiber, sodium, and total protein. A positive RERI signals attenuation of protection if either of the stratum-specific hazard ratios is less than 1, while a negative RERI indicates synergistic protection when both stratum-specific hazard ratios are less than 1.


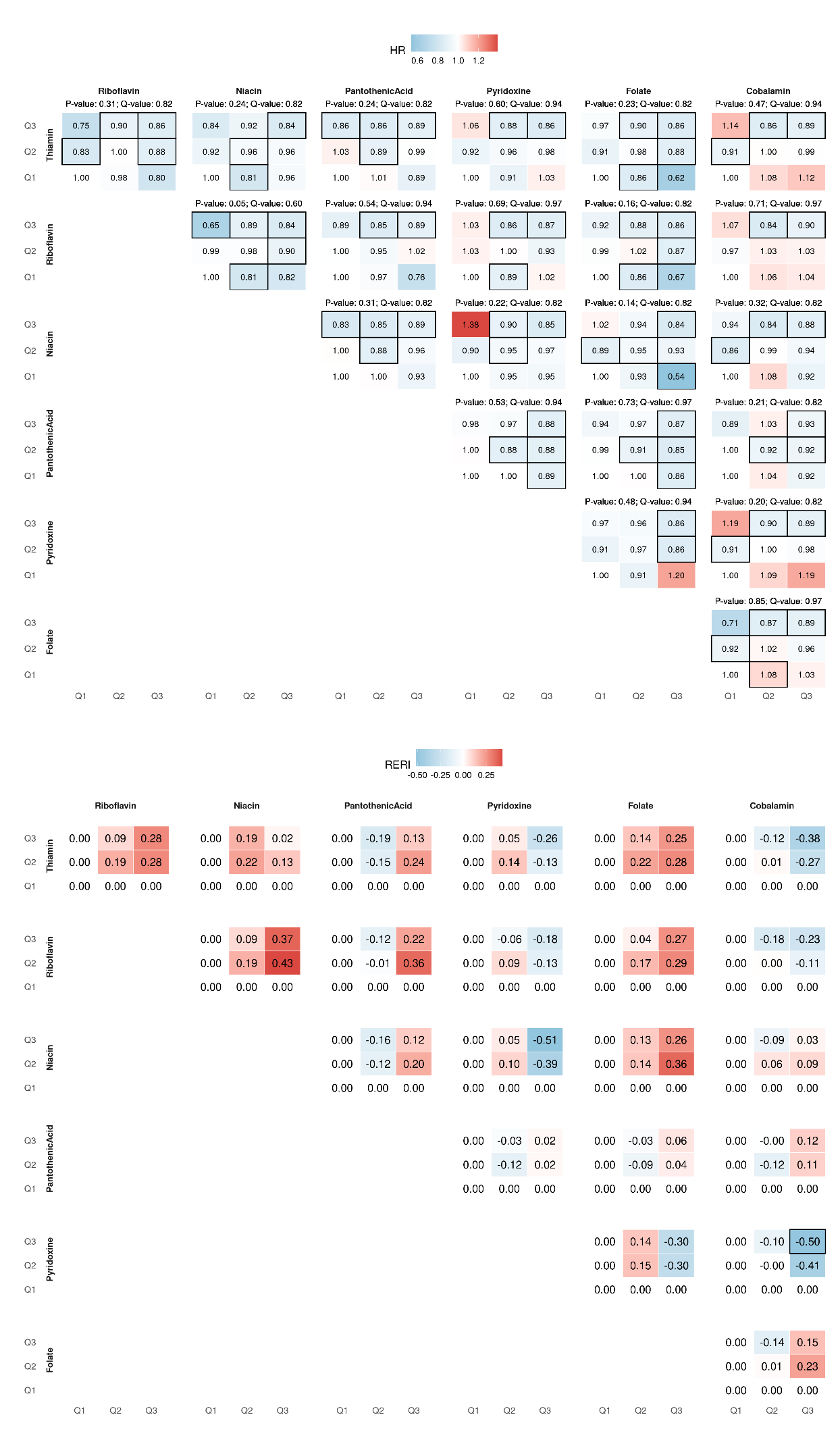


Figure S9. Associations of long-term intakes of individual B-vitamins with incident stroke in the Women’s Health Initiatives, stratified by baseline subgroups. Models were adjusted for age, ethnicity, income, education, smoking, drinking, MET-mins per week, family history of stroke, BMI group, abdominal obesity, hypertension, ever use of lipid-lowering drugs, time-weighted average intakes of total energy, vitamins C, D, E, α- and β-carotene, selenium, magnesium, potassium, calcium, dietary fiber, sodium, and total protein. CI: confidence interval; HR: hazard ratios.


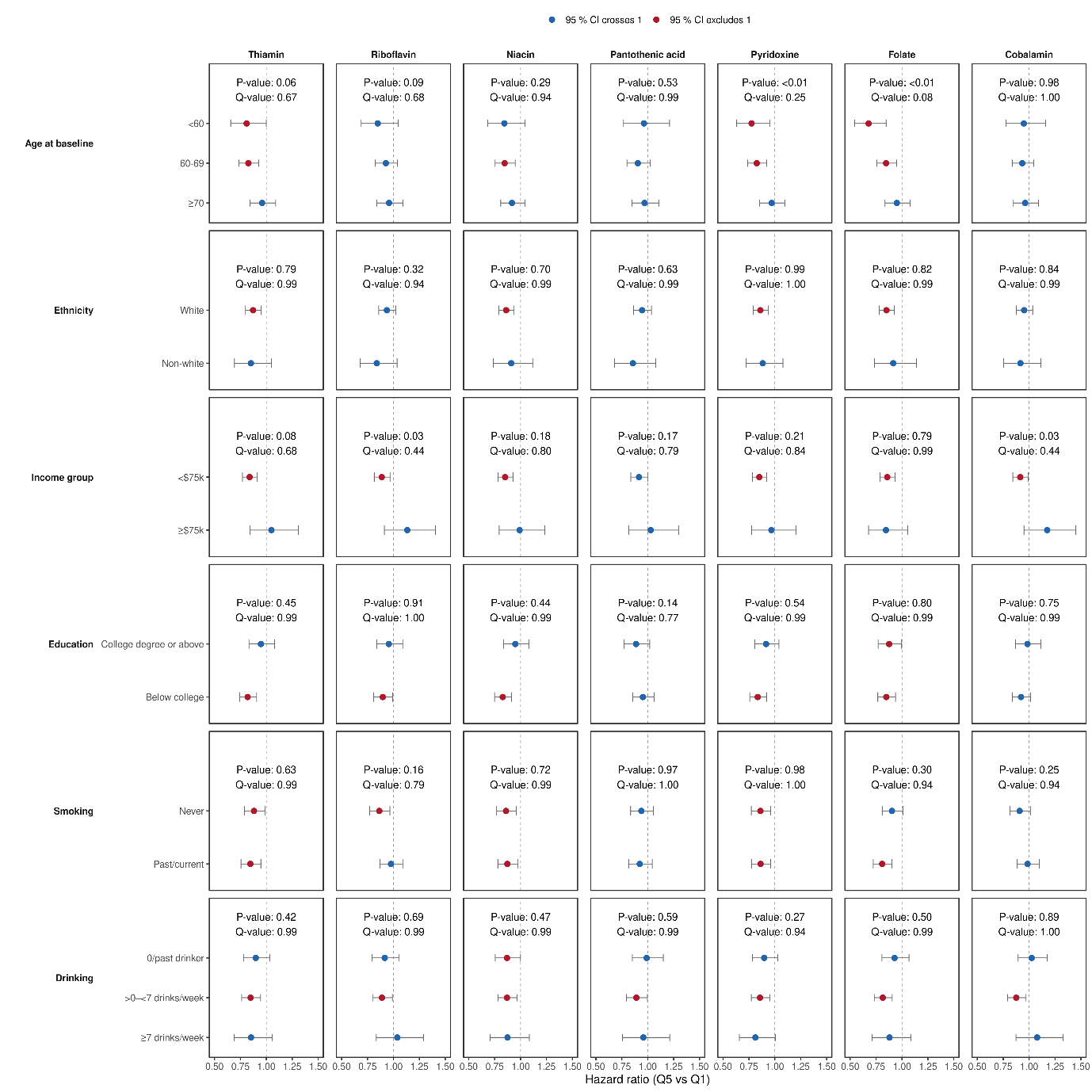

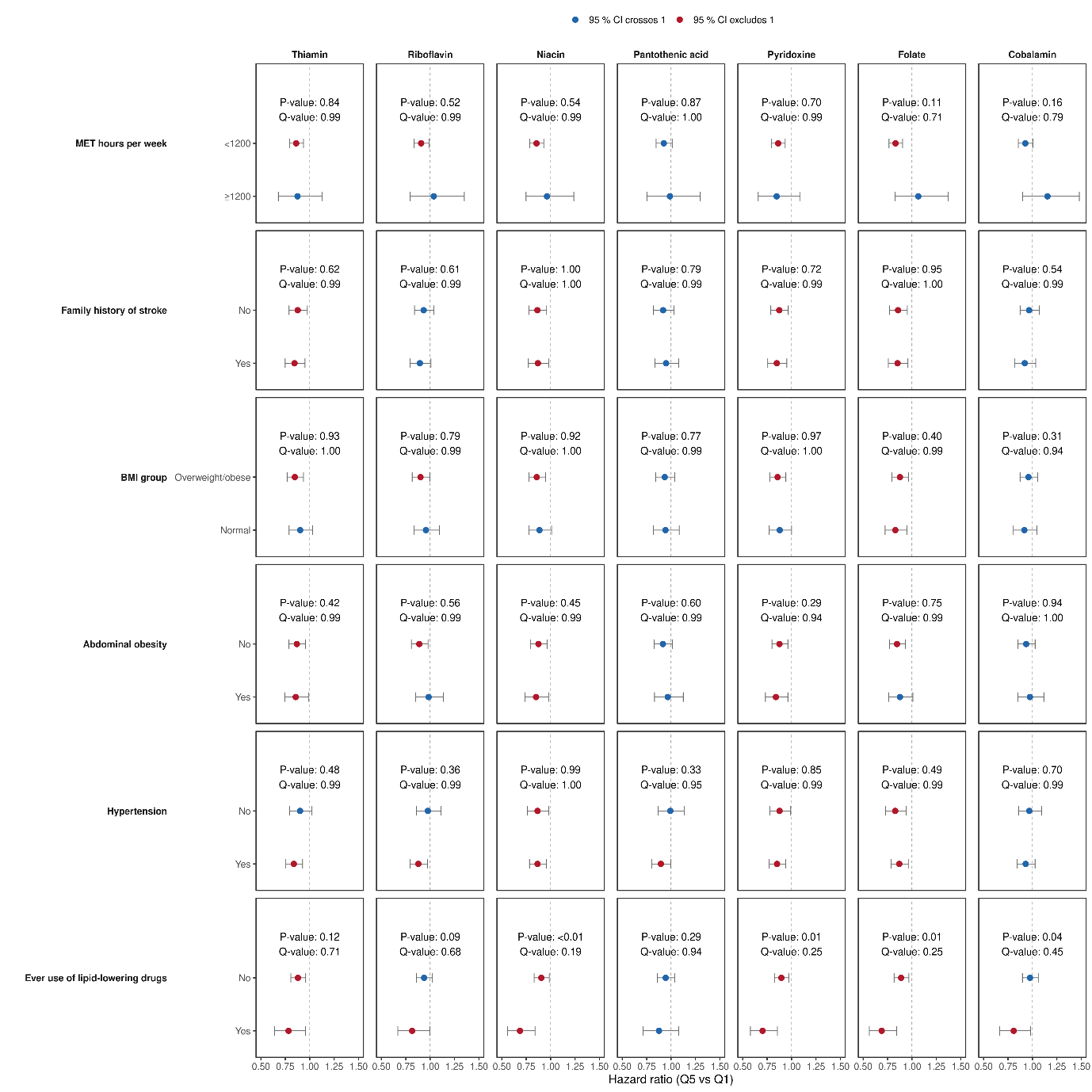


Figure S10. Associations of circulating concentrations of individual B-vitamins with incident stroke in the All of US Research Program, stratified by baseline subgroups. Models were adjusted for age, sex, ethnicity, education, smoking, drinking, family history of stroke, BMI group, abdominal obesity, hypertension, ever use of oral glucose-lowering drugs, and ever use of lipid-lowering drugs at baseline. CI: confidence interval; HR: hazard ratios.


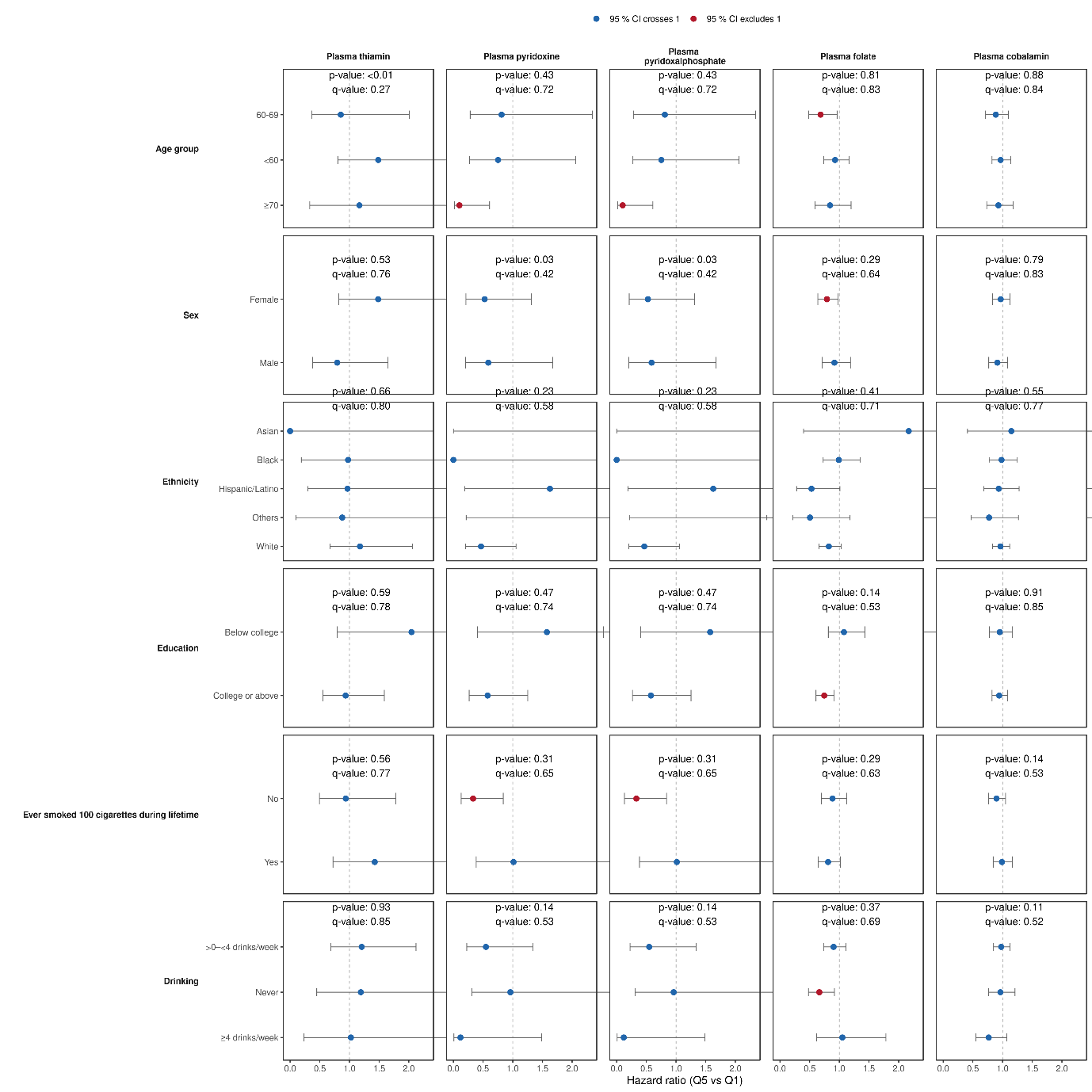

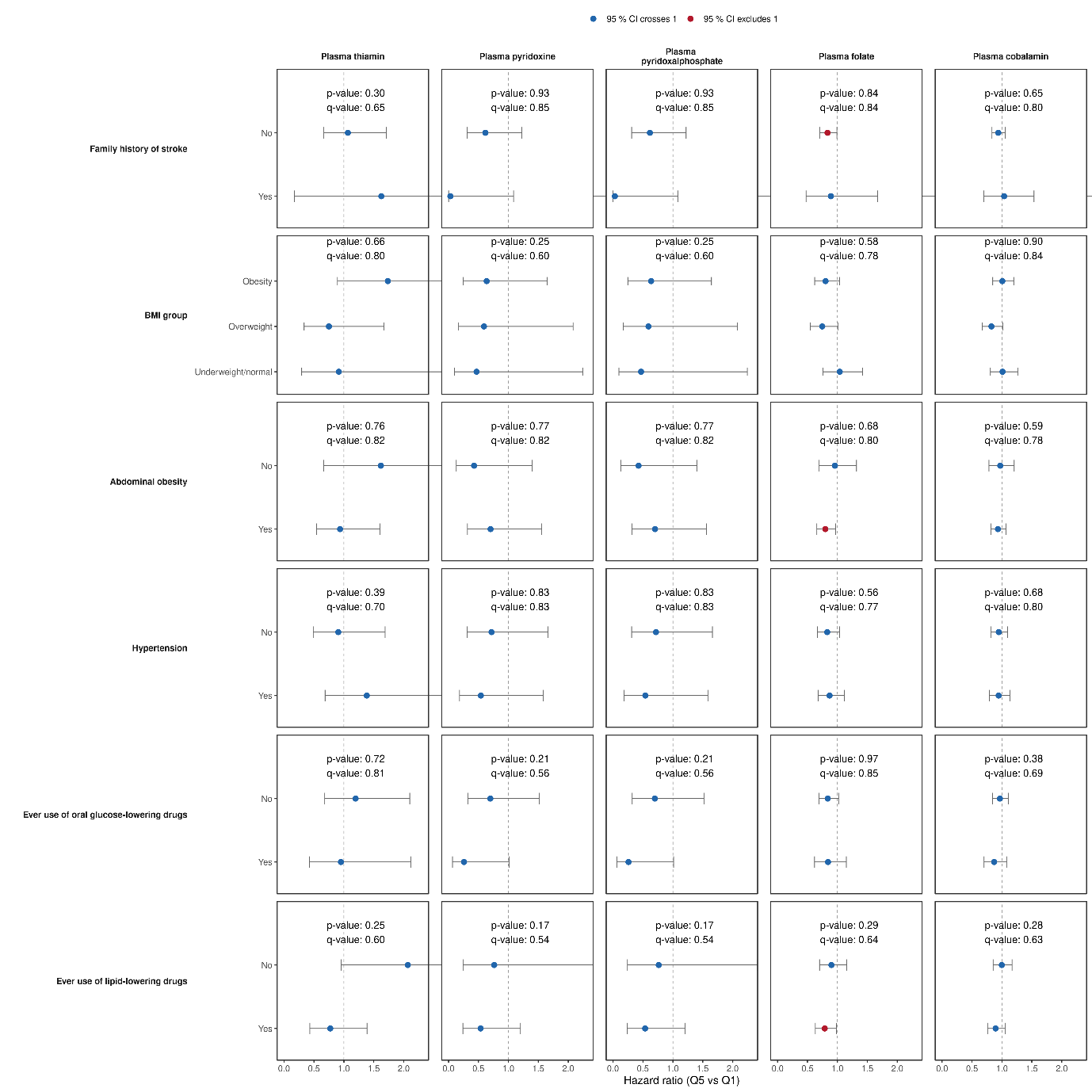


Figure S11. Associations of long-term intakes of individual B-vitamins with incident stroke in the Women’s Health Initiatives, restricting to non-users of multivitamin supplement. A: Dose-response associations of time-weighted average intakes of individual B-vitamins with incident stroke in the Women’s Health Initiative. B: Comparisons of stroke risk across quintiles of time-weighted average intakes of individual B-vitamins, with the lowest quintile as the reference. C: Comparisons of stroke risk across quintiles of baseline intakes and subsequent changes in individual B-vitamins. Models were adjusted for age, ethnicity, income, education, smoking, drinking, MET-mins per week, family history of stroke, BMI group, abdominal obesity, hypertension, ever use of lipid-lowering drugs at baseline, time-weighted average intakes of total energy, vitamins C, D, E, α- and β-carotene, selenium, magnesium, potassium, calcium, dietary fiber, sodium, and total protein. CI: confidence interval; DFE: dietary folate equivalent; HR: hazard ratios.


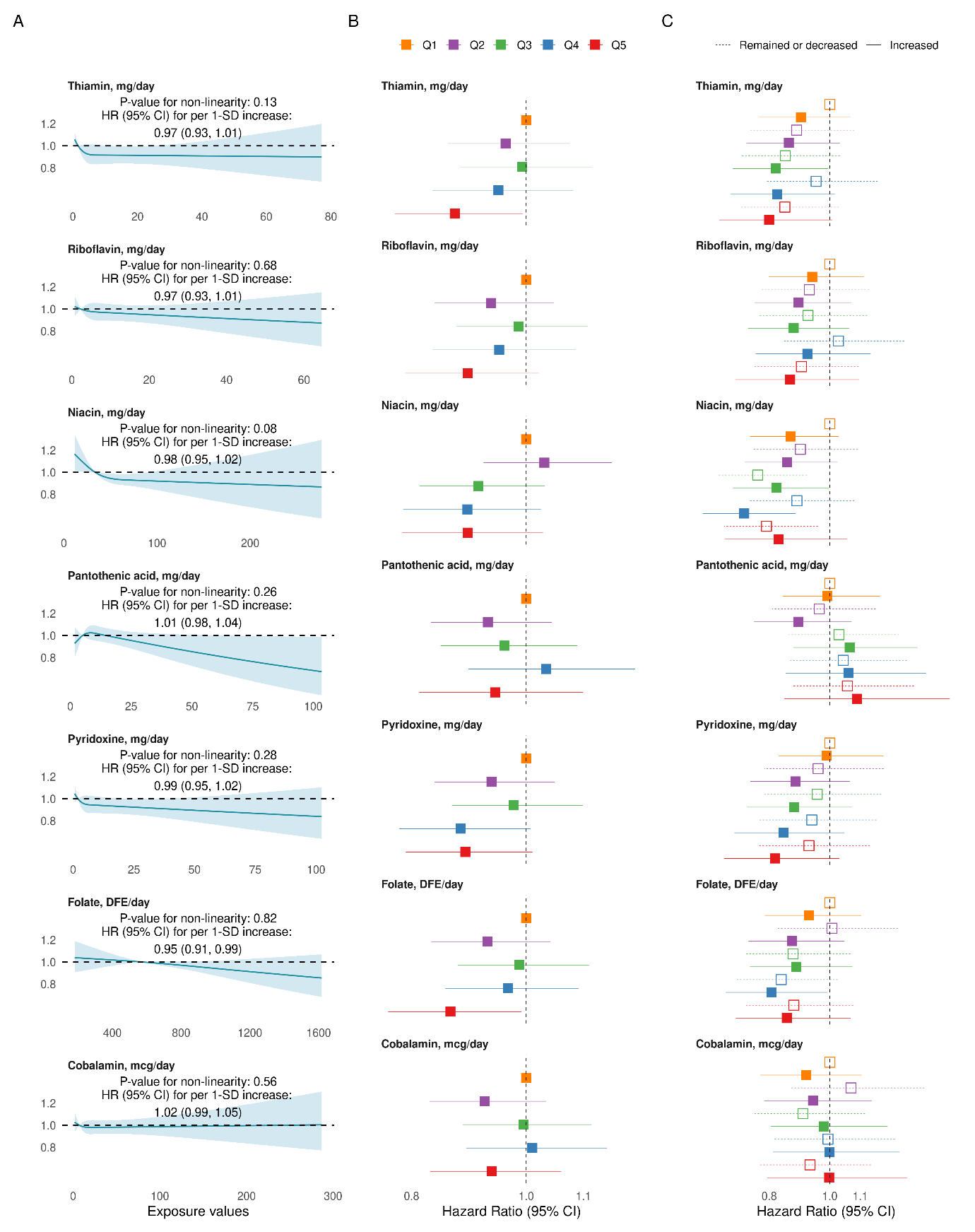


Figure S12. Sensitivity analysis of Fine-Gray models to avoid bias from the competing risk of death in Women’s Health Initiatives. Models were adjusted for age, ethnicity, income, education, smoking, drinking, MET-mins per week, family history of stroke, BMI group, abdominal obesity, hypertension, ever use of lipid-lowering drugs, time-weighted average intakes of total energy, vitamins C, D, E, α- and β-carotene, selenium, magnesium, potassium, calcium, dietary fiber, sodium, and total protein. CI: confidence interval; HR: hazard ratios.


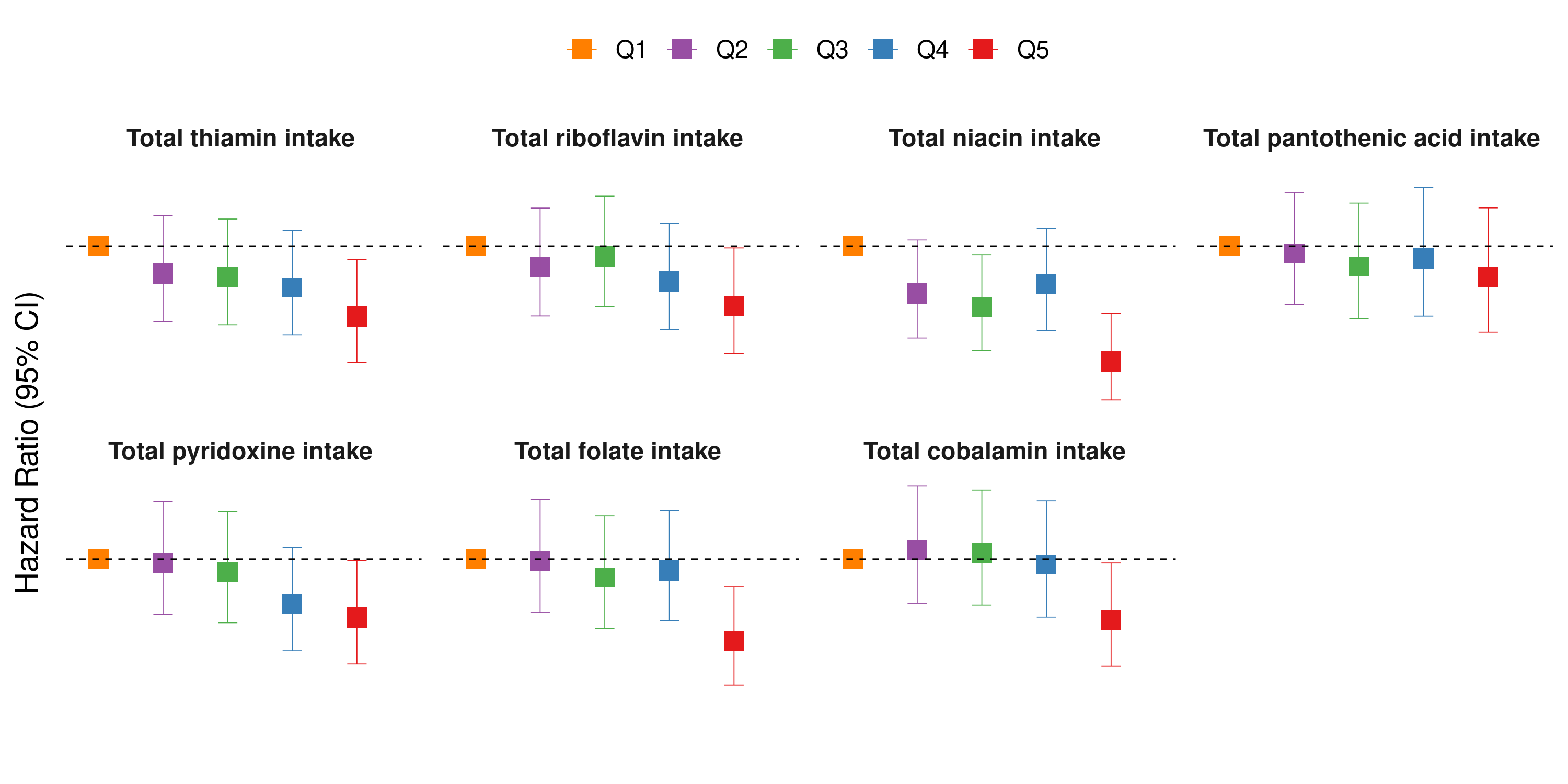


Figure S13. Sensitivity analysis of Fine-Gray models to avoid bias from the competing risk of death in the All of US Research Program. Models were adjusted for age, sex, ethnicity, education, smoking, drinking, family history of stroke, BMI group, abdominal obesity, hypertension, ever use of oral glucose-lowering drugs, and ever use of lipid-lowering drugs at baseline. CI: confidence interval; HR: hazard ratios.


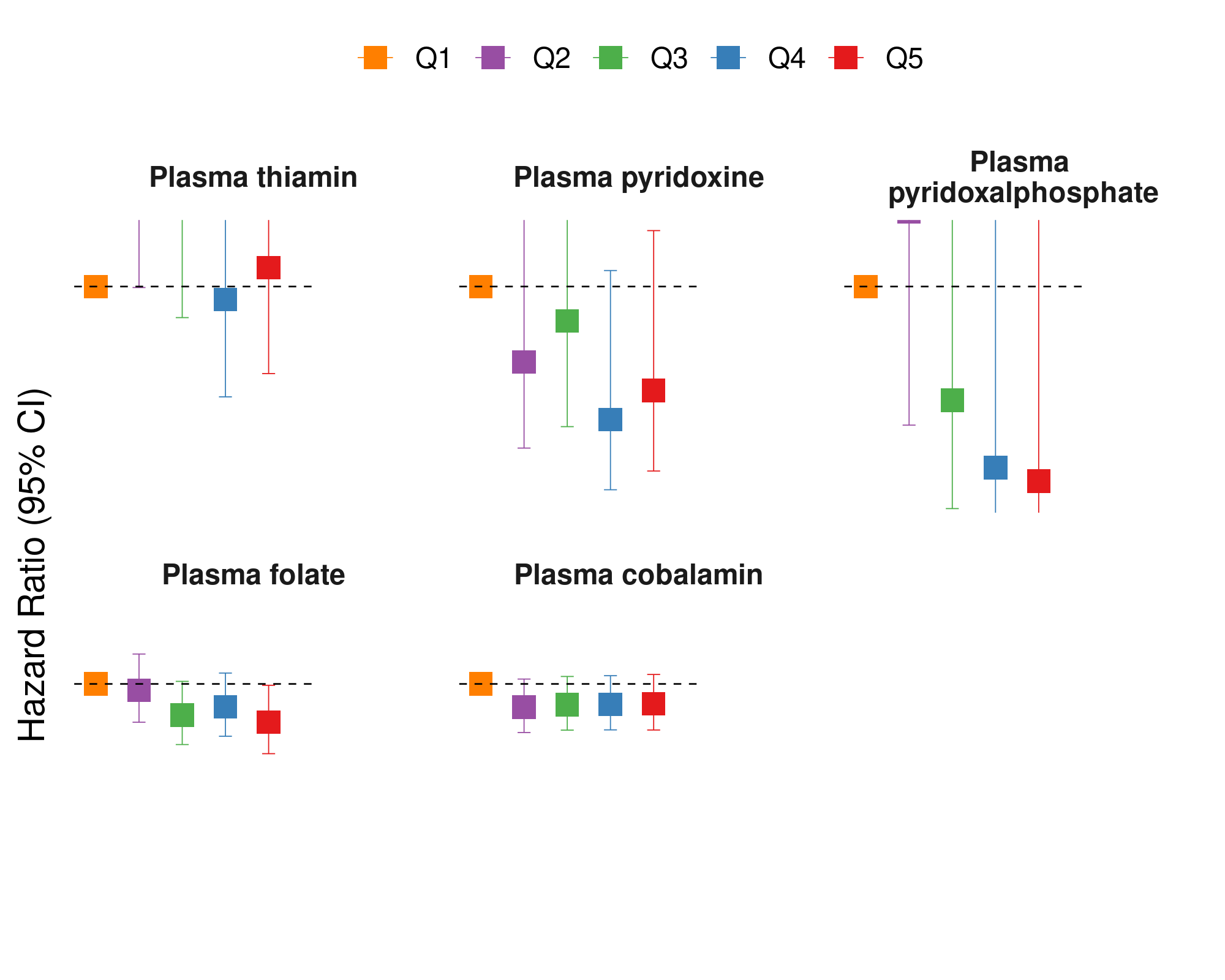

Supplement: Supplementary file 1 [file mmc1.docx]
